# Paleozoic Protein Fossils Illuminate the Evolution of Vertebrate Genomes and Transposable Elements

**DOI:** 10.1093/molbev/msac068

**Published:** 2022-03-28

**Authors:** Martin C. Frith

**Affiliations:** 1 Artificial Intelligence Research Center, AIST, Tokyo, Japan; 2 Graduate School of Frontier Sciences, University of Tokyo, Chiba, Japan; 3 Computational Bio Big-Data Open Innovation Laboratory, AIST, Tokyo, Japan

**Keywords:** pseudogene, exaptation, transposon, retrotransposon, paleovirology

## Abstract

Genomes hold a treasure trove of protein fossils: Fragments of formerly protein-coding DNA, which mainly come from transposable elements (TEs) or host genes. These fossils reveal ancient evolution of TEs and genomes, and many fossils have been exapted to perform diverse functions important for the host’s fitness. However, old and highly degraded fossils are hard to identify, standard methods (e.g. BLAST) are not optimized for this task, and few Paleozoic protein fossils have been found. Here, a recently optimized method is used to find protein fossils in vertebrate genomes. It finds Paleozoic fossils predating the amphibian/amniote divergence from most major TE categories, including virus-related Polinton and Gypsy elements. It finds 10 fossils in the human genome (eight from TEs and two from host genes) that predate the last common ancestor of all jawed vertebrates, probably from the Ordovician period. It also finds types of transposon and retrotransposon not found in human before. These fossils have extreme sequence conservation, indicating exaptation: some have evidence of gene-regulatory function, and they tend to lie nearest to developmental genes. Some ancient fossils suggest “genome tectonics,” where two fragments of one TE have drifted apart by up to megabases, possibly explaining gene deserts and large introns. This paints a picture of great TE diversity in our aquatic ancestors, with patchy TE inheritance by later vertebrates, producing new genes and regulatory elements on the way. Host-gene fossils too have contributed anciently conserved DNA segments. This paves the way to further studies of ancient protein fossils.

## Introduction

Genomes contain relics of formerly protein-coding DNA, which may be functionless and neutrally evolving, or in some cases have gained new, nonprotein-coding functions. Most of them are derived from either transposable elements or host genes.

Transposable elements (TEs) are parasitic, or perhaps symbiotic, DNA elements that get copied or moved from one genome location to another. They have often proliferated greatly, so that for example the human genome has millions of TE-derived segments comprising at least ∼50% of the genome. Most of these segments are highly mutated fragments, no longer active TEs.

TEs have had a massive impact on the evolution of their hosts (Warren et al. 2015; Etchegaray et al. 2021). They cause mutations by their proliferation, and also by ectopic recombination among TE copies, causing deletions, inversions, and duplications. This can duplicate or inactivate genes (Barsh et al. 1983; Hayakawa et al. 2001), or change their tissue-specific expression (Ting et al. 1992). Some host genes have evolved from TEs, such as the vertebrate RAG genes that generate the diverse antibodies and T-cell receptors of the immune system (Kapitonov and Koonin, 2015), and syncytin genes that seem to enable cell fusion in placental development (Dupressoir et al. 2005). Some DNA elements that regulate gene expression have also evolved from TEs (Ting et al. 1992; Jordan et al. 2003).

A series of studies in 2006–2007 found thousands of TE-derived nonprotein-coding elements with strong evolutionary conservation in mammals (Bejerano et al. 2006; Kamal et al. 2006; Nishihara et al. 2006; Xie et al. 2006; Gentles et al. 2007; Lowe et al. 2007). They often occur in gene deserts, and nearest to developmental genes (Lowe et al. 2007). These TE insertions often predate the placental/marsupial divergence (Mesozoic), but few clearly predate the mammal/bird divergence (Paleozoic), and an exceptional handful (“at least several”) were shown to predate the amniote/amphibian divergence (Bejerano et al. 2006). It is thus remarkable that a later study claimed to find 133 TE insertions predating the divergence of humans and ray-finned fish, by comparing human TE fragments found by RepeatMasker to vertebrate genome alignments (Lowe and Haussler, 2012).

The boundary between TEs and viruses is blurry, and an entire field, paleovirology, is mainly based on viral insertion fossils in eukaryote genomes (Barreat and Katzourakis, 2022). The oldest viral fossils found so far seem to be Mesozoic (Suh et al. 2014; Barreat and Katzourakis, 2022).

TEs are diverse and their classification is partly arbitrary (Kojima, 2019; Storer et al. 2021), but eukaryotic TEs are conventionally split into *retrotransposons* which duplicate by reverse transcription of their RNA into DNA, and *DNA transposons* which do not. Major types of retrotransposon are: LINEs (long interspersed nuclear elements), LTR retrotransposons (which bear long terminal repeats), YR (tyrosine recombinase) retrotransposons, and Penelope-like elements. These are further subclassified, for example LINEs have clades and sub-clades such as Hero, Nimb, L1, I, and CR1. Major types of DNA transposon are: DDE transposons (named after three key amino acids in the transposase), Cryptons (YR transposons), Helitrons, and Polintons (also called Mavericks). These are also subdivided, for example DDE transposons have “superfamilies” such as Academ, hAT, Kolobok, and piggyBac. Finally, nonautonomous TEs such as short interspersed nuclear elements (SINEs) typically encode no proteins, and propagate by hijacking enzymes from autonomous TEs.

Many types of TE have *patchy* presence across host genomes, meaning that a TE type is present in distantly related hosts but absent in some closer relatives of those hosts (Yuan and Wessler, 2011; Chalopin et al. 2015). This can sometimes be explained by ordinary vertical inheritance, with multiple losses of the TE family (Fawcett and Innan, 2016). Contrarily, it has been suggested that long-term vertical persistence of TEs may be rare, so their long-term persistence depends on horizontal transfer (Gilbert and Feschotte, 2018). Thus, in order to understand the evolution of TE families in eukaryotes, it is valuable to know what TE types were present in ancestral eukaryotes (Fawcett and Innan, 2016).

Host-gene-derived protein fossils are often called “pseudogenes.” They usually arise from duplication of (part of) a gene, such that one of the two copies is either not expressed or dispensable so evolves away from its protein-coding ancestry. Many such duplications are created by reverse-transcription of mRNA to DNA (e.g. by retrotransposon enzymes), producing intron-depleted fossils termed “processed pseudogenes.” There are also nonduplicated “unitary pseudogenes,” for example the *GULO*/*GULOP* gene/pseudogene for making vitamin C, which is nonfunctional in primates and guinea pigs (Nishikimi et al. 1994).

Some pseudogenes seem to have significant functions, for example by being transcribed into an antisense RNA regulator of its cognate gene (Korneev et al. 1999), or regulating transcription (Huang et al. 2017), or generating small interfering RNAs (Tam et al. 2008). The Xist RNA involved in X chromosome inactivation has evolved partly from a formerly protein-coding gene, and partly from TEs (Elisaphenko et al. 2008). The boundary between protein fossils and functional protein-coding genes is fuzzy: a decaying gene such as *GULO* may produce peptides whose contribution to the organism’s fitness fluctuates around zero, in the process of gene death or resurrection (Brosius and Gould, 1992; Cheetham et al. 2020).

Genetic fossils are often found by comparing a genome to a database of TE or gene sequences (Harrison, 2021; Storer et al. 2021). This can be done by either DNA-to-DNA or DNA-to-protein comparison. Protein-coding DNA tends to evolve by changes that preserve the encoded amino acids or replace them with similar ones: thus highly diverged sequences can be detected more effectively at the protein level (States et al. 1991). On the other hand, protein fossils evolve without amino-acid conservation. Thus, new TE families are often found by protein-level matches to distantly related families, whereas relics of known TE families are best detected by DNA-level matches to a model approximating the family’s most-recent active ancestor. RepeatMasker files of such DNA-level matches are available for many genomes (Smit et al. 2015).

Protein-level matches have usually been sought with BLAST (Altschul et al. 1997), which is not optimized for fossils. Central to sequence matching methods are parameters defining the (dis)favorability of substitutions and gaps, which provide the definition of similarity. BLAST uses a 20 × 20 amino-acid substitution matrix (BLOSUM or PAM), which is based on substitution rates in living proteins, so is likely suboptimal for fossils.

Therefore, we recently developed a new DNA-to-protein matching method, which allows frameshifts within matches (Yao and Frith, 2021), implemented in LAST (https://gitlab.com/mcfrith/last). Its main advantage is that it sets the substitution, gap, and frameshift parameters by maximum-likelihood fit to given sequence data. It uses a richer 64 × 21 substitution matrix, allowing for example preferred matching of asparagine (encoded by aac or aat) to agc than to tca, which both encode serine. It judges homology based on not just one alignment, but on many alternative ways of aligning the putative homologs. This proved more sensitive than BLAST for finding human TE protein fossils, and for the first time it found YR retrotransposon fossils in the human genome (Yao and Frith, 2021).

Here, this method is used to find new protein fossils in human and slowly evolving Lagerstätte genomes: alligator, turtle, coelacanth (a lobe-finned fish closely related to land vertebrates), and chimera (a nonbony cartilaginous fish); and also frog due to its intermediate phylogenetic position (table 1). The number of new fossils is relatively small, but they are especially ancient and include types of TE not found in human before. They thus illuminate the evolutionary history of TE content, and reveal strongly conserved ancient exaptations, including of host-gene fossils.

**Table 1.** Genome Versions and TE Protein Fossils.

Organism		Genome Assembly	RepeatMasker	TE	Of Which	
		(from NCBI or UCSC)	Version (source)	Fossils	Novel^a^	(%)
Human	*Homo sapiens*	UCSC hg38.analysisSet	4.0.7 (UCSC)	546,821	1,641	(0.3)
Alligator	*Alligator mississippiensis*	ASM28112v4	4.0.6 (NCBI)	410,092	46,065	(11)
Turtle	*Chrysemys picta bellii*	Chrysemys_picta_bellii-3.0.3	4.0.6 (NCBI)	430,459	63,301	(15)
Frog	*Xenopus tropicalis*	UCB_Xtro_10.0	4.0.8 (NCBI)	135,507	14,837	(11)
Coelacanth	*Latimeria chalumnae*	UCSC latCha1	4.0.5 (rmsk^b^)	286,944	279,710	(97)
Chimaera	*Callorhinchus milii*	UCSC calMil1	4.0.3 (UCSC)	105,995	31,098	(29)

a Not found by this version of RepeatMasker.

b repeatmasker.org.

## Results and Discussion

### Protein Fossil-Finding Pipeline

For each organism, homologous segments were found between the genome and a set of protein sequences comprising TE proteins from RepeatMasker plus proteins encoded by host genes of that organism. When multiple homologies overlapped in the genome, only the strongest was kept, to avoid homologies between different types of TE or between TEs and host genes. Homologies overlapping annotated protein-coding segments of the genome were removed. Finally, host-gene homologies were discarded if they overlapped TEs annotated by RepeatMasker: this removes true-but-unwanted homologies due to host-gene protein-coding segments that evolved from, for example SINEs. The resulting fossils, including a genome browser hub, are available at https://github.com/mcfrith/protein-fossils.

The homology search used a significance threshold of one expected random match to the whole set of proteins per 10^9^ bp, so there would be ∼3 matches in total between the human genome and all the proteins, if the sequences were perfectly random. However, naive matching would find many nonhomologous similarities of “simple sequences” such as atatatatatatatat: these were suppressed with tantan (Frith, 2011; Yao and Frith, 2021). The false-positive rate was estimated by comparing the reversed (but not complemented) human genome to the whole set of proteins, producing 19 spurious matches in total.

### New TE Fossils

For the organisms analyzed in this study, the number of TE protein fossils found per genome ranges from ∼100,000 to ∼500,000, most of which correspond to known TE fragments in public RepeatMasker files (table 1). The human genome has especially few new TE fossils, indicating how thoroughly human TEs have been analyzed. The coelacanth fossils are almost all new relative to the RepeatMasker annotations, simply because those annotations have very few TE types, illustrating that TE analysis is lacking for some genomes at any snapshot in time (Sotero-Caio et al. 2017).

### Classifying Unknown Repeats

**Fig. 1.**
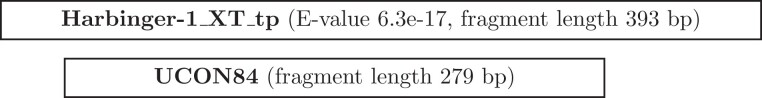
Overlap between a TE protein fossil (upper box) and a repeat of unknown type (lower box) in the alligator genome (at coordinate 15,466,729 in NW_017707593.1).

RepeatMasker genome annotations include repeats of unknown type, which might not be TEs (Bao et al. 2015; Smit et al. 2015). In alligator and turtle (but not the other genomes), some of these unknown repeats could be classified based on large and consistent overlaps with TE protein fossils (table 2). One of these repeats, UCON84, also occurs in the human genome: it is derived from a DDE transposon in the PIF/Harbinger superfamily (fig. 1). The UCON84 consensus sequence, obtained from Dfam (Storer et al. 2021), has shorter and weaker (but significant) homology to PIF/Harbinger proteins (not shown). The consensus is expected to approximate an ancestral sequence and thus have clearer homology, but it is hard to make an accurate consensus of ancient fragments.

**Table 2.** Classifying Unknown Repeats in Alligator and Turtle.

Unknown Repeat	TE Type
REP-2_CPB	CR1 (LINE)
REP-3_CPB	L2 (LINE)
REP-6_CPB	CR1 (LINE)
REP-22_CPB	hAT-Tag1
REP-28_CPB	CR1 (LINE)
REP-31_CPB	Gypsy (LTR)
SAT-928_Crp	Penelope
UCON84	PIF/Harbinger

### Inter-Genome Homology

**Fig. 2.**
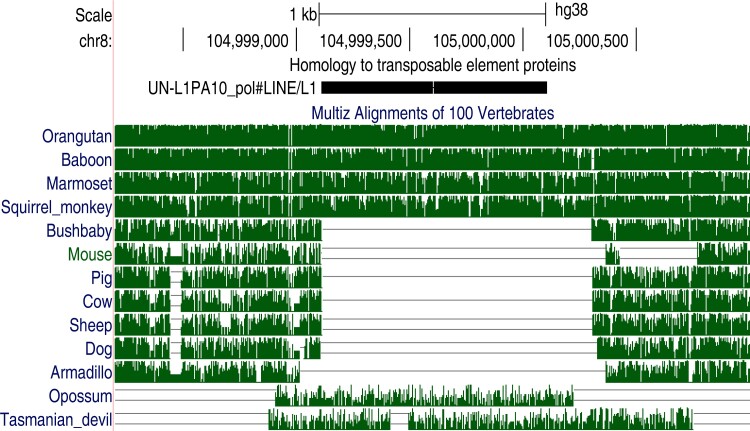
A TE protein fossil in human chromosome 8, with confusing inter-genome homology. Black bar near top: alignment of an L1 LINE protein. Green tracks: alignments between the human and other genomes. Screen shot from http://genome.ucsc.edu.

The age of genetic fossils can be inferred by comparing different genomes. For example, figure 2 shows a human TE fossil aligned to an L1 LINE protein, alongside mammal genome alignments from the UCSC genome browser (Kent et al. 2002; Harris, 2007). This L1 insertion is present in ape and monkey genomes but absent from bushbaby and other placental mammals, showing that the insertion occurred in a common ancestor of simians after their divergence from strepsirrhine primates. It is thus curious that the L1 insert is aligned to two marsupial genomes: opossum and tasmanian devil. Marsupials also have L1s, and these marsupial regions are indeed annotated as L1s by RepeatMasker. Thus, these human and marsupial *inserts* are true homologs, because all L1s share common ancestry, but the *insertions* are not homologous: not descended from a common ancestral insertion. The inserts might even be orthologs, if their common ancestor is no older than the placental/marsupial divergence.

Why, then, do these marsupial alignments extend into flanking sequence beyond the insert? It is hard to determine the precise endpoint of homology between distantly related sequences: alignments overshoot or undershoot. These human-marsupial alignments were made with the HoxD55 substitution matrix and gap parameters that are prone to large overshoots (Frith et al. 2008).

**Fig. 3.**
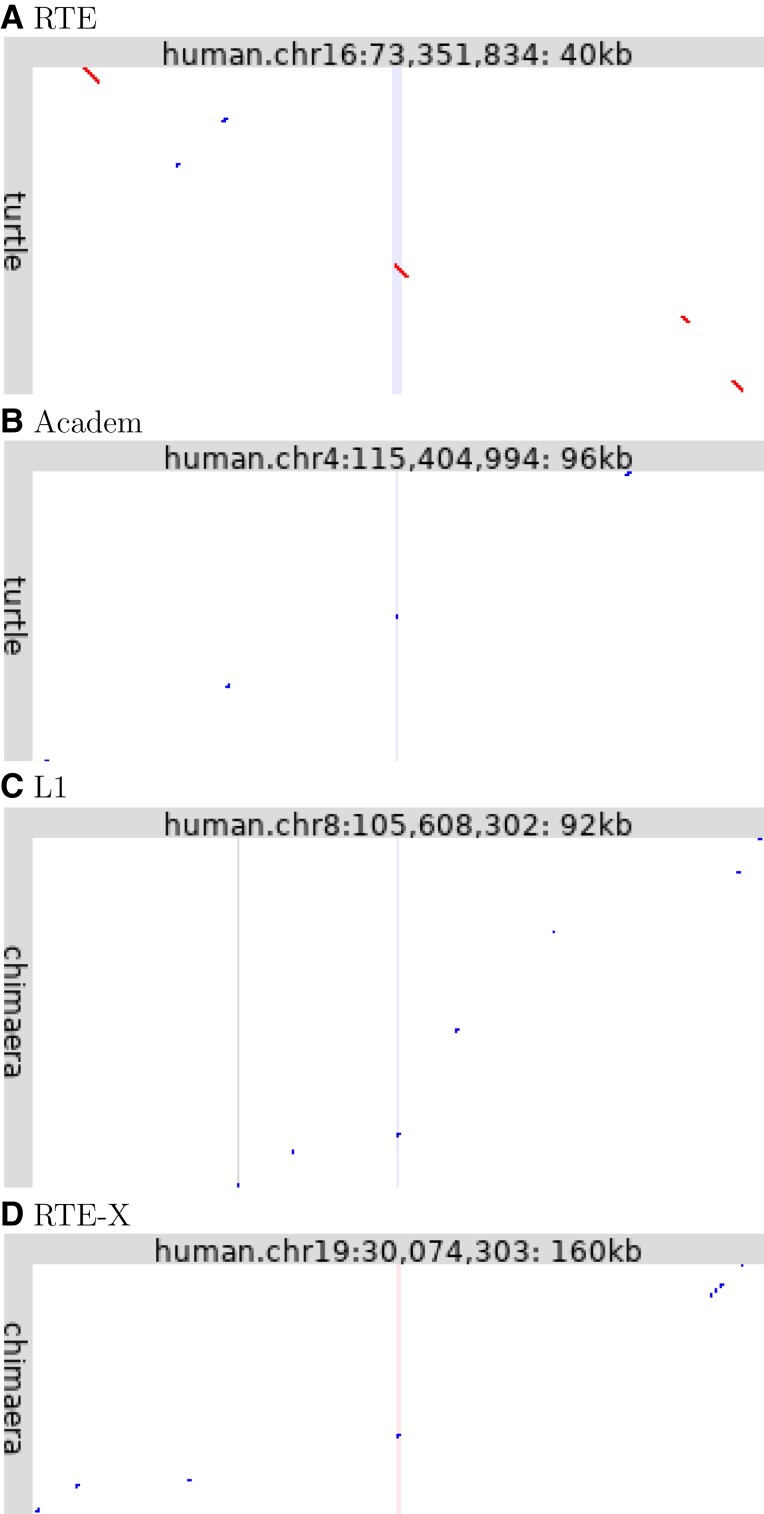
Ancient conserved TE insertions. Each panel shows alignments between part of the human genome (horizontal) and turtle (*A*,*B*) or chimera (*C*,*D*). Red dots indicate same-strand alignments, blue dots opposite-strand alignments. The central vertical lines show the location in human of the TE fossil (pink: forward strand, blue: reverse strand). The vertical gray line in panel *C* shows a protein-coding exon of *ZFPM2*.

For this study, new pair-wise genome alignments were made, by finding homologous regions (Frith and Noé, 2014) and cutting them down to most-similar one-to-one alignments (Frith and Kawaguchi, 2015). This tends to find higher-similarity alignments than those from UCSC and elsewhere, indicating that a higher fraction of the alignments are orthologous (Frith and Kawaguchi, 2015). This probably does not avoid nonhomologous TE insertions, so a new step was added: isolated alignments were discarded, by only keeping groups of alignments that are nearby in both genomes. Some examples are in figure 3: each panel shows one TE fossil in the human genome (central vertical stripe) that overlaps an inter-genome alignment (diagonal lines/dots). The alignments are not isolated: they are flanked by other alignments, indicating homology of not just the TE insert but also the flanking regions. Because these are distantly related genomes, most of the DNA lacks similarity and is unaligned. The alignable fragments are probably conserved by natural selection.

A possible objection is that these examples might be independent insertions of an abundant TE into homologous regions of two genomes. This cannot be ruled out, but the key point is that these alignments are not only homologies but most-similar one-to-one homologies: it would be a strong coincidence for these single-best matches to independently be in homologous regions.

### TE Types Newly Found in Human

**Fig. 4.**
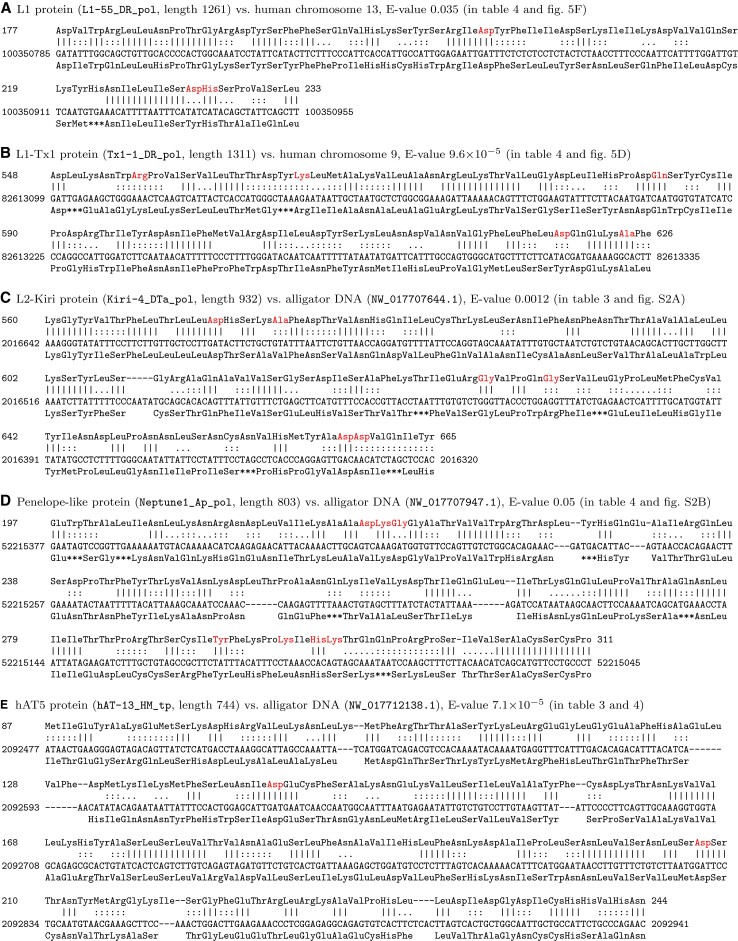
Alignments between TE proteins and DNA. The DNA’s translation is shown below it, with *** for stop codons. ||| indicates a match, ::: a positive substitution score, and … a zero substitution score. Red color indicates: (*A*) conserved residues in L1 EN domains (Moran and Gilbert, 2002), (*B*,*C*) conserved residues in LINE RT domains (Malik et al. 1999), (*D*) conserved residues in Penelope-like elements (Arkhipova, 2006), (*E*) catalytic hAT residues (Atkinson, 2015). The start coordinates are 1-based, whereas the coordinates in tables 3 and 4 are 0-based (so they differ by 1). This figure was made with maf-convert from the LAST package.

The human TE protein fossils include several types of TE that have not been found in human before (table 3). These are all LINEs or DDE transposons, and are in addition to the first human YR retrotransposons (DIRS and Ngaro) and first-but-one Polintons we recently reported (Yao and Frith, 2021). Some were found directly in human, others were found in another genome and mapped to human via the inter-genome alignments (“found in” column). The E-value indicates significance/confidence of the DNA–protein homology: it is the expected number of times to find such a similarity between the whole genome and the entire set of proteins, if they were random sequences. Some of the E-values are quite high, indicating lower confidence. On the other hand, most of these putative DNA–protein homologies overlap human/nonmammal genome alignments, which would be a strong coincidence if they were random similarities (fig. 3). These DNA–protein alignments often cover conserved signature amino acids of the TE, which are not always conserved in the fossils, as expected if they have lost protein-coding function (fig. 4, supplementary fig. S1).

**Table 3.** TE Protein Fossils of Types Newly Found in Human (all detected instances of these types).

Type	Aligned Protein	Chromosome	Start	Length (bp)	Nearest Gene	Intergene	Intron	Found In	E-value	Age
						Length (kb)				
*Retrotransposons*										
I	I-1_DR_pol	3	139,262,204	119	*MRPS22*	299		Alligator	0.026	Amniote
I	I-1_DR_pol	9	32,218,473	204	*ACO1*	3,171		Turtle	0.97	Amniote
Nimb	Nimb-1_DR_pol	2	4,986,057	216	*SOX11*	1,844		Alligator	0.027	Amniote
Nimb	Nimb-2_SSa_pol	3	70,169,802	144	*MDFIC2*	226		Turtle	0.014	Amniote
Nimb	Nimb-2_DR_pol	10	76,759,130	141	*KCNMA1*	309		Alligator	1.6	Amniote
Nimb	Nimb-2_LG_pol	13	53,319,346	227	*OLFM4*	4,089		Human	3.1e − 05	Tetrapod
Nimb	Nimb-12_DR_pol	X	7,635,163	193	*VCX*	488		Alligator	0.00012	Amniote
Nimb	Nimb-6_DR_pol	X	87,287,424	258	*KLHL4*	685		Human	5.6e − 14	Amniote
Nimb	Nimb-12_LMi_pol	X	87,289,312	87	*KLHL4*	685		Human	2.5	Tetrapod
L2-Daphne	Daphne-3_OL_pol	15	76,184,366	282	*TMEM266*		16	Alligator	5.8e − 05	Amniote
L2-Kiri	Kiri-3_HMM_pol	3	157,982,382	158	*SHOX2*	592		Turtle	0.012	Amniote
L2-Kiri	Kiri-1_DTa_pol	16	53,518,165	255	*AKTIP*	95		Turtle	0.053	Amniote
L2-Kiri	Kiri-4_DTa_pol	18	25,027,321	256	*ZNF521*	582		Alligator	0.0012	Amniote
R2-Hero	HEROTn	2	118,705,507	284	*EN1*	731		Alligator	0.48	Amniote
R2-Hero	HERO-2_BF_pol	4	13,163,078	145	*RAB28*	1,939		Alligator	1.3e − 06	Amniote
R2-Hero	HERO-2_BF_pol	6	72,553,710	317	*KCNQ5*	219		Turtle	6.5e − 05	Tetrapod
R2-Hero	HEROTn	7	36,766,591	241	*AOAH*	128		Alligator	6.9e − 06	Amniote
R2-Hero	HERO-2_BF_pol	8	71,079,407	240	*EYA1*	461		Turtle	0.064	Amniote
R2-Hero	HEROTn	8	76,862,008	444	*ZFHX4*		7	Alligator	4.4e − 12	Amniote
R2-Hero	HERO-1_SP_pol	11	91,081,544	376	*CHORDC1*	2,002		Human	0.0099	Amniote
R2-Hero	HEROTn	14	53,387,314	538	*DDHD1*	796		Alligator	3.2e − 09	Amniote
R2-Hero	HEROTn	15	67,559,478	285	*MAP2K5*		13	human	3.4e − 06	Amniote
R2-Hero	HEROTn	X	31,319,778	294	*DMD*		57	Human	2.3e − 08	Amniote
RTE	RTE-2_LVa_pol	1	88,450,891	352	*PKN2*	1,335		Human	0.74	—
RTE	RTE-4_LCh_pol	1	216,744,731	223	*ESRRG*		82	Human	0.2	Amniote
RTE	RTE-2_LVa_pol	2	198,403,329	316	*PLCL1*	1,120		Human	0.0067	Amniote
RTE	RTE-2_LVa_pol	2	204,082,119	410	*ICOS*	584		Human	2.2e − 13	Amniote
RTE	RTE-12_SP_pol	3	67,685,630	136	*SUCLG2*	337		Alligator	5e − 05	Amniote
RTE	RTE-12_SP_pol	3	169,026,953	209	*MECOM*	988		Alligator	0.0053	Amniote
RTE	RTE1_Mars_pol	3	172,228,179	190	*FNDC3B*		21	Human	0.026	—
RTE	RTE-4_LCh_pol	10	33,939,004	87	*PARD3*	775		Turtle	3.3e − 12	Amniote
RTE	UN-72133877_Spu_pol	10	82,724,195	249	*NRG3*		317	Alligator	2e − 07	Amniote
RTE	RTE-4_LCh_pol	13	58,819,808	129	*DIAPH3*	1,936		Alligator	0.029	Amniote
RTE	RTE-2_LVa_pol	16	73,371,834	433	*ZFHX3*		138	Human	9.3e − 16	Amniote
RTE	RTE-4_CPB_pol	X	97,057,978	283	*DIAPH2*		108	Human	0.07	—
Rex1/Babar	REX1-1_BF_pol	4	34,782,619	209	*ARAP2*	4,919		Turtle	5.2e − 13	Amniote
Rex1/Babar	Rex1-24_NV_pol	4	130,232,079	245	*C4orf33*	4,033		Alligator	0.019	Amniote
*DNA Transposons*										
Academ	Academ-1_NV_tp	4	115,452,994	152	*NDST4*	1,970		Turtle	0.0067	Amniote
EnSpm	EnSpm-1_CGi	2	129,063,904	314	*HS6ST1*	1,661		Turtle	0.0008	Amniote
EnSpm	EnSpm-11_HM	2	180,729,359	142	*UBE2E3*	973		Alligator	0.04	Amniote
EnSpm	EnSpm-11_HM	3	180,734,783	182	*CCDC39*	233		Human	2	Amniote
Ginger1	Ginger1-10_HM_tp	3	14,7687,919	166	*ZIC1*	1,281		Alligator	1.2e − 20	Amniote
hAT19	hAT-39_LCh_tp	1	3,746,554	188	*CCDC27*	16		Coelacanth	1.5	Sarcopterygian
hAT19	hAT-31_CPB_tp	2	104,030,381	203	*POU3F3*	2,036		Human	7.6e − 08	—
hAT19	hAT-39_LCh_tp	2	143,799,848	418	*ARHGAP15*	170		Human	3.2e − 21	Amniote
hAT19	hAT-31_CPB_tp	4	34,434,122	613	*ARAP2*	4,919		Human	5.1e − 11	—
hAT19	hAT-31_CPB_tp	7	57,524,151	625	*ZNF716*	6,572		Human	3.8e − 14	—
hAT19	hAT-13_LCh_tp	16	75,903,914	276	*CPHXL*	551		Alligator	3.5e − 11	Amniote
hAT19	hAT-31_CPB_tp	20	26,187,394	444	*ZNF337*	5,561		Human	3.1e − 08	—
hAT5	hAT-13_HM_tp	18	38,666,092	430	*CELF4*	4,389		Alligator	7.1e − 05	Tetrapod

**Table 4.** Pre-tetrapod TE Protein Fossils Found in Human (all detected instances).

Type	Aligned Protein	Chromosome	Start	Length (bp)	Nearest Gene	Intergene	Intron	Found In	E-value	Age
						Length (kb)				
*Retrotransposons*										
CR1	CR1-4_LCh_pol	4	13,161,246	107	*RAB28*	1,939		Alligator	1.9e − 06	Tetrapod
CR1	UN-BfCR1_pol	4	111,235,163	187	*PITX2*	1,503		Chimera	0.0078	Gnathostome
CR1	CR1-1_CM_pol	5	94,809,895	122	*MCTP1*		69	Alligator	0.0049	Tetrapod
CR1	HER_LINE_pol	6	98,370,405	277	*POU3F2*	1,551		Turtle	0.022	Tetrapod
CR1	CR1-1_CPB_pol	16	78,813,330	608	*WWOX*		779	Turtle	0.022	Tetrapod
CR1	CR1-4_LCh_pol	18	72,559,688	232	*CBLN2*	198		Alligator	9.9e − 08	Tetrapod
Nimb	Nimb-2_LG_pol	13	53,319,346	227	*OLFM4*	4,089		Human	3.1e − 05	Tetrapod
Nimb	Nimb-12_LMi_pol	X	87,289,312	87	*KLHL4*	685		Human	2.5	Tetrapod
L1	L1-2_LCh_pol	3	70,416,318	138	*MDFIC2*	642		Coelacanth	0.61	Gnathostome
L1	L1-3_LCh_pol	8	105,654,302	154	*ZFPM2*		154	Human	1	Gnathostome
L1	L1-5_LCh_pol	9	2,050,774	284	*SMARCA2*		7	Human	0.093	Tetrapod
L1	L1-55_DR_pol	13	100,350,784	171	*PCCA*		28	Human	0.035	Gnathostome
L1	L1-42_DR_pol	18	55,784,184	514	*TCF4*	961		Alligator	6e − 07	Tetrapod
L1-Tx1	Tx1-1_DR_pol	9	82,613,098	237	*RASEF*	984		Human	9.6e − 05	Gnathostome
L1-Tx1	Tx1-5_CGi_pol	10	129,519,611	156	*MGMT*		69	Coelacanth	3e − 16	Gnathostome
L2	CR1-41_DR_pol	5	166,711,049	95	*TENM2*	3,554		Turtle	0.4	Tetrapod
L2	CR1-9_DR_pol	6	45,881,206	149	*CLIC5*	347		Alligator	0.032	Tetrapod
L2^a^	L2-13_DRe_pol	7	108,869,078	298	*DNAJB9*	2,088		Human	2.9e − 25	Tetrapod
L2-Crack	Crack-11_BF_pol	1	216,137,121	109	*USH2A*		77	Alligator	3.5e − 08	Tetrapod
L2-Crack	Crack-1_SSa_pol	5	109,502,097	189	*PJA2*	280		Human	0.00013	Tetrapod
R2-Hero	HERO-2_BF_pol	6	72,553,710	317	*KCNQ5*	219		Turtle	6.5e − 05	Tetrapod
RTE-X	RTEX-16_SK_pol	19	30,154,303	438	*ZNF536*	209		Human	0.00078	Gnathostome
Penelope	Neptune1_Ap_pol	4	187,183,800	258	*FAT1*	1,272		Alligator	0.05	Tetrapod
Penelope	Penelope-2_CPB_pol	13	106,429,488	180	* EFNB2*	999		Human	0.00017	Tetrapod
Gypsy	Gypsy-14_SSa_1p	1	38,669,733	195	*RRAGC*	791		Human	5.9e − 11	Tetrapod
Gypsy	Gypsy-37_CGi_1p	2	57,598,618	325	*VRK2*	1,521		Alligator	8.3e − 12	Tetrapod
Gypsy	Gypsy-13_CPB_1p	19	30,111,148	159	*URI1*	209		Human	0.017	Tetrapod
Gypsy	Gypsy-24_XT_1p	X	98,972,571	328	*PCDH19*	2,687		Human	4.6e − 23	Tetrapod
DIRS	DIRS-21A_XT_pol	3	55,911,374	235	*ERC2*		62	Alligator	1.1e − 08	Tetrapod
DIRS	DIRS-1a_Amnio_pol	9	13,728,242	223	*NFIB*	802		Human	0.0037	Tetrapod
DIRS	DIRS-7_NV_pol	9	20,189,423	234	*MLLT3*	553		Turtle	0.86	Tetrapod
DIRS	DIRS-9_NV_pol	10	16,734,086	144	*RSU1*		57	Alligator	0.84	Tetrapod
DIRS	DIRS-5B_LCh_2p	16	78,152,629	218	*WWOX*		49	Turtle	3.2e − 17	Tetrapod
*DNA Transposons*										
PIF/Harbinger	Harbinger-3_LCh_tp	2	145,279,820	206	*ZEB2*	3,324		Human	4.4e − 05	Tetrapod
PIF/Harbinger	Harbinger3_DR_tp	2	176,676,027	180	*MTX2*	875		Human	0.0012	Tetrapod
hAT-Blackjack	hAT-38_LCh_tp	7	14,272,601	191	*DGKB*		160	Alligator	6.5e − 10	Tetrapod
hAT-Tip100	HAT-3_BF_tp	4	4,696,650	164	*STX18*	317		Alligator	0.0021	Tetrapod
hAT-Tip100	UN-Zaphod1_Ola_tp	4	129,428,560	225	*C4orf33*	4,033		Human	1.8e − 11	Tetrapod
hAT19	hAT-39_LCh_tp	1	3,746,554	188	*CCDC27*	16		Coelacanth	1.5	sarcopterygian
hAT5	hAT-13_HM_tp	18	38,666,092	430	*CELF4*	4,389		Alligator	7.1e − 05	Tetrapod
Crypton-A	CryptonA-1_OL_yr	12	14,468,609	174	*ATF7IP*		9	Alligator	2.2e − 70	Gnathostome
Polinton	Polinton-1_Crp_px	3	114,555,204	205	*ZBTB20*		83	Human	2.8	Tetrapod
Polinton	Polinton-1_AMi_atp	20	52,577,239	269	*ZFP64*	781		Turtle	4.4e − 15	Tetrapod
Polinton	Polinton-1_DR_px	20	55,516,705	163	*CBLN4*	1,346		Turtle	7.4e − 11	Tetrapod

^a^Previously found by RepeatMasker.

**Fig. 5.**
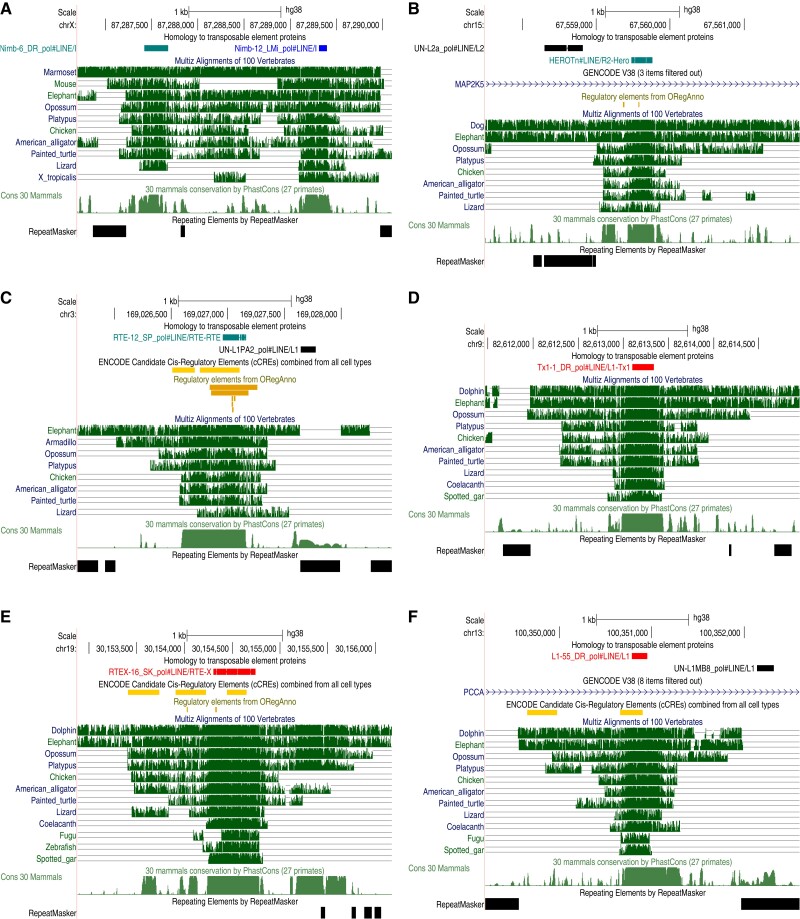
Ancient conserved TE insertions in the human genome. Each panel shows, from top to bottom: TE protein fossils, alignments of the human genome to other vertebrate genomes, evolutionary conservation in mammals (phastCons), and repeats found by RepeatMasker. Some panels also show annotations of regulatory elements and Gencode genes (introns). Screen shots from http://genome.ucsc.edu.

Genomic data show ancient conservation and exaptation of these fossils (fig. 5, supplementary fig. S2). It can be seen that they lie in human genome regions conserved in nonmammals, and are not annotated by RepeatMasker. These regions have strong evolutionary conservation in mammals according to phastCons (Siepel et al. 2005), independent of their conservation in nonmammals. Some of these fossils overlap candidate regulatory elements or known transcription factor binding sites (Lesurf et al. 2016; Moore et al. 2020): the Hero fossil in figure 5*B* overlaps a CEBPB binding site, and the RTE fossil in figure 5*C* overlaps binding sites for GATA2, STAT1, JUND, FOS, and JUN. Figure 5*A* shows two Nimb fragments that coincide with conserved DNA segments: presumably they come from one Nimb insertion, which predates the amniote/amphibian divergence. (Only one of these Nimb fragments is aligned to frog: the other may be deleted or not detected in frog.)

These fossils clarify the historical presence of TE types in vertebrates. They make presence of several TE types less patchy among vertebrates, thus explicable by vertical inheritance rather than horizontal transfer. Nimb-type LINEs have been found in insects, mollusks, teleost (bony) fish (Kapitonov et al. 2009; Chalopin et al. 2015), and turtle (Smit et al. 2015): here Nimb relics are found from ancient tetrapods, and also in coelacanth. This makes the presence of Nimb in vertebrates less patchy, and suggests vertical inheritance from the common ancestor of bony vertebrates. The Hero clade was found in sea urchin, lancelet, and fish (Kojima and Fujiwara, 2004; Kapitonov et al. 2009): its presence in ancient tetrapods fits with vertical inheritance from deuterostome ancestors. Hero LINEs are unusual in having a restriction-like endonuclease (Kojima and Fujiwara, 2004), unlike all other human LINE relics except Mam_R4. The I clade was previously found in fish and some invertebrates (Kapitonov et al. 2009): here hundreds are found in turtle and a few in alligator. Daphne was previously found in sea urchin and arthropods (Schön and Arkhipova, 2006), plus lancelet and zebrafish (Smit et al. 2015): here 67 fragments are found in coelacanth, 6 in chimera, 6 in turtle, and 4 in alligator, rounding out its historical presence in vertebrates. The Rex1/Babar clade has been found patchily in nonsarcopterygian fish excluding chimera, plus frog and lizard (Chalopin et al. 2015; Smit et al. 2015): here it is found in ancestral amniotes and also coelacanth and chimera, rendering its distribution nonpatchy.

RepeatMasker distinguishes two types of RTE-like LINE: BovB and RTE; it finds only BovB in human, whereas it finds RTE in turtle and zebrafish. Previous reports of RTE in human seem to be BovB elements that were not classified separately (Kojima, 2018). This study finds RTEs from amniote ancestors, and thousands in coelacanth, again suggesting vertical inheritance from ancestors of bony vertebrates.

These fossils also provide support for TE origin of some genes. Ginger1 transposons were previously found in some invertebrates including lancelet (Bao et al. 2010), but not in sarcopterygians (Yuan and Wessler, 2011; Chalopin et al. 2015). Their relics are found here in alligator, turtle, coelacanth, and many in frog. This makes it more plausible that the human *GIN1* gene was indeed exapted from Ginger1 in ancestral amniotes (Bao et al. 2010). At least one pre-amniote Ginger1 relic was also exapted for nonprotein-coding function (table 3). Similarly, hAT19 fossils from amniote ancestors support the hAT19 origin of the amniote-specific gene *CGGBP1* (Yellan et al. 2021), which binds CGG repeats and regulates gene expression (Singh and Westermark, 2015). hAT19 fragments have been exapted for nonprotein-coding functions too.

In the four tetrapod genomes just one hAT5 fragment is found, which is conserved in all of them: the single exapted relic of an ancient hAT5 infection (fig. 4*E*). hAT5 was previously found in some invertebrates (Putnam et al. 2007) and fish (Smit et al. 2015), and is unusual in having 5 bp TSDs (target site duplications), whereas all previously known hATs have 8 bp TSDs (Putnam et al. 2007).

### Anciently Conserved TE Fossils

The human genome contains diverse TE protein fossils that are older than the amniote/amphibian divergence (table 4). It is striking that they include nearly all major types of TE: LINEs, Penelope-like elements, LTR retrotransposons (Gypsy), YR retrotransposons (DIRS), DDE transposons, a Crypton, and Polintons. Eight of them (seven LINEs and a Crypton) are shared by human and chimera, making them older than the last common ancestor of all jawed vertebrates. Three of these oldest fossils are shown in figure 5*D–F*: their ancient exaptation is supported by their conserved presence in mammal, reptile, and bony-fish genomes, their strong conservation in mammals (phastCons), and sometimes by evidence of regulatory function.

A further 882 TE protein fossils that predate the mammal/reptile divergence were found in the human genome (table 5). Most of these (745, 84%) are novel (not annotated by RepeatMasker), as are all but one of the pre-tetrapod fossils (table 4). These ancient TE fossils are often in megabase-scale gene deserts or large ($∼10^{5}$ bp) introns (tables 3 and 4). The nearest genes are significantly enriched in developmental functions such as nervous system development, cell morphogenesis, and axonogenesis (PANTHER GO overrepresentation test, Mi et al. 2021). Some other types of TE protein fossil in the human genome were never found to predate the mammal/reptile divergence: these are strikingly less diverse, just ERVs (endogenous retroviruses) and a handful of DNA transposon superfamilies (table 6).

**Table 5.** Other Pre-amniote TE Protein Fossils in Human.

Type	Number	Of Which Not New^a^
*Retrotransposons*		
CR1	204	92
Dong-R4	1	0
Vingi	1	0
L1	137	22
L1-Tx1	12	0
L2	167	14
L2-Crack	25	1
BovB	28	1
RTE-X	4	0
Penelope	54	1
Gypsy	83	3
DIRS	37	0
Ngaro	28	0
*DNA Transposons*		
Kolobok-T2	1	0
PIF/Harbinger	18	1
PiggyBac	7	0
TcMar-Mariner	1	0
TcMar-Pogo	2	0
TcMar-Tc1	4	0
TcMar-Tigger	5	0
hAT-Ac	7	1
hAT-Blackjack	12	1
hAT-Charlie	6	0
hAT-Tip100	21	0
Crypton-A	3	0
Polinton	14	0

a Previously found by RepeatMasker.

**Table 6.** TE Types in Human Never Found to be Pre-amniote.

Type	Number	Of Which Not New^a^
*Retrotransposons*		
ERV1	17,653	16,951
ERVK	2,188	2,114
ERVL	22,541	21,076
ERVL-MaLR	19,140	18,111
*DNA Transposons*		
MULE-MuDR	437	374
Merlin	37	35
TcMar-Tc2	783	751
hAT-Tag1	205	202
Helitron	23	21

a Previously found by RepeatMasker.

These TE relics from ancient vertebrates help us to understand the ancestral mobilome, which has been difficult, especially since TEs might have been horizontally transferred (Chalopin et al. 2015). For example, it has been suggested that mammal L1s were introduced by horizontal transfer into a common ancestor of therian (live-bearing) mammals (Ivancevic et al. 2018). We now have direct evidence that L1-like TEs were present in a common ancestor of jawed vertebrates, and hundreds of L1 fragments predate the amniote divergence (table 5). Actually, repeatmasker.org lists 353 L1 fragments in the platypus genome (ornAna1), so perhaps L1s were vertically inherited by mammals, but became inactive early in the monotreme lineage. There are also L1-Tx1 fossils from gnathostome ancestors (table 4): this supports the suggestion that L1 clades including Tx1 diverged in a common ancestor of mammals and fish (Ichiyanagi et al. 2007), which was not certain since Tx-like L1s are prone to horizontal transfer between marine hosts (Ivancevic et al. 2018).

For other TE types too—DIRS, Polinton, and PIF/Harbinger—their previously noted patchiness among tetrapods (Chalopin et al. 2015) is explained by ancient loss of activity, since they were present in tetrapod ancestors. The emerging picture is that ancient vertebrates had many diverse types of TE, like present-day teleost fish but unlike mammals or birds (Chalopin et al. 2015).

The pre-amniote BovB fossils (table 5) are particularly informative, because BovB has frequently been horizontally transferred (Ivancevic et al. 2018). Interestingly, the phylogeny of BovB elements differs greatly *but not entirely* from the phylogeny of their host organisms: amniote BovBs are all in a central branch of the tree and fish BovBs on outer branches (Ivancevic et al. 2018, fig. 2A). Knowing that BovBs were present in amniote ancestors, it seems likely that BovB initially entered amniotes by vertical inheritance, perhaps specifically into squamate reptiles, before being horizontally transferred among amniotes and arthropod vectors.

Regarding LTR retrotransposons, it is intriguing that ancient Gypsy-like fossils are found (tables 4 and 5), but ancient ERV (endogenous retrovirus) fossils are not (table 6). The origin of vertebrate retroviruses has been debated (Hayward, 2017): ERVs may have evolved from Gypsy-like elements in a common ancestor of amniotes and amphibians (Hellsten et al. 2010).

The Crypton relic in *ATF7IP* (table 4) was found in a previous study (Kojima and Jurka, 2011), which showed that it inserted in a common ancestor of amniotes, and found a similar sequence in chimera. We can now push the age of this insertion back to the gnathostome ancestor (supplementary fig. S3). This is a similar age to other Crypton insertions that became protein-coding regions of vertebrate genes, including *KCTD1* which is closely related to the *ATF7IP* Crypton (Kojima and Jurka, 2011). This suggests that active Cryptons may have been present in our ancestors only before the gnathostome divergence and not since. The *ATF7IP* Crypton has an intact open reading frame in some nonmammal vertebrates (Kojima and Jurka, 2011), including alligator and chimera (supplementary figs. S4–5): so it may have been exapted as a protein-coding sequence in gnathostome ancestors and lost function in mammals.

**Fig. 6.**
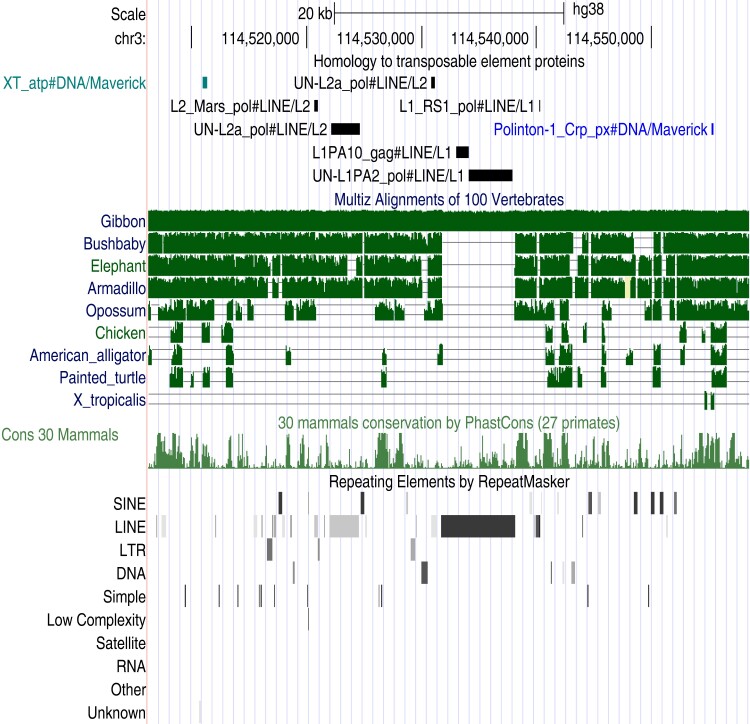
Ancient Polinton/Maverick fragments in an intron of *ZBTB20* on human chromosome 3. The two fragments are colored blue-green and blue, with younger TE fossils in between (black).

The age of the oldest Polinton insertions is greatly increased from 95 million years (Barreat and Katzourakis, 2021) to ∼350 million years (the amniote/amphibian divergence). This age is inferred from homologous polinton fragments in (e.g.) turtle and frog, which are flanked by other turtle–frog homologies (supplementary figs. S6–7). So either these polintons independently inserted into homologous regions of amniote and amphibian genomes, or, more parsimoniously, they come from insertion in a common ancestor of amniotes and amphibians. Ancient insertion is also implied by human polinton relics that align to a wide range of mammals and amniotes in the UCSC genome alignments (fig. 6).

These protein fossils might be much younger than their insertions, if the intact TE benefits host fitness so remains intact (i.e. protein coding) by natural selection of the host. Intact TEs are usually thought not to benefit host fitness, but intact Polintons might protect the host from viruses, in particular iridoviruses that infect cold-blooded vertebrates (Barreat and Katzourakis, 2021). Nevertheless, the human Polinton relics are no longer intact, yet some have strong phastCons conservation in mammals indicating exaptation.

### Conserved RepeatMasker Fossils

For sake of comparison, the age of previously known TE fossils (from RepeatMasker) was inferred in the same way. RepeatMasker includes many more TE fossils, especially nonprotein-coding SINEs. It is tuned to have a false-positive fraction of 0.2% (Hubley et al. 2016), which corresponds to $∼10^{4}$ false hits in the human genome. There are 133 RepeatMasker hits in human that are conserved in frog, of which 84 (63%) are especially ancient types of repeat: UCON, Eulor, LFSINE, and AmnSINE1 (Bejerano et al. 2006; Nishihara et al. 2006; Gentles et al. 2007). Most of these are unknown types of repeat, and may not be TEs. In contrast, there are 73 RepeatMasker hits in human that are conserved in coelacanth, which are not obviously enriched in ancient repeat types. They include primate-specific L1P and SVA elements, which are surely false-positive RepeatMasker annotations. A few may be real, but it is hard to know which ones or have confidence in them. Unfortunately, RepeatMasker files do not state the significance (E-value) of each hit. In summary, the oldest confident minimum age for previously known TE insertions (apart from TE-derived genes) is the amniote/amphibian divergence (Bejerano et al. 2006).

This casts doubt on the previously reported TE insertions predating the human/teleost divergence (Lowe and Haussler, 2012). Aside from false RepeatMasker hits, that study mentioned no countermeasures for nonhomologous insertions (fig. 2).

The tetrapod TEs found here (table 4) are almost completely disjoint from previously known ones: the latter are mostly unknown repeat types or SINEs. The newly found LINEs might be the autonomous counterparts of the ancient SINEs, in particular, AmnSINE1 was thought to be mobilized by an undiscovered L2-like LINE (Nishihara et al. 2006).

### Genome Tectonics

Sometimes, two TE fossils of the same type lie strikingly near each other in the human genome. An example is in figure 6: two Polinton relics are separated by 44 kb, which is remarkably close considering there are only 40 Polinton fragments in the genome. They might come from two independent insertions into a Polinton hotspot, but a simpler explanation is that they come from one Polinton, and drifted apart due to younger TE insertions between them. It is well known that old TEs get fragmented by younger insertions, but it is interesting to consider how far apart they can drift. If there is a locally higher rate of insertion than deletion, this might over time produce large introns and gene deserts. Ancient fossils can be markers of such long-term rifting. Among the pre-amniote TE fossils, there are a few hundred such pairs separated by 30–3,000 kb.

### Host-Gene-Derived Protein Fossils

This study found 27,240 host-gene-derived protein fossils in the human genome, of which 4,303 (16%) are new: not in Gencode V37 or RefSeq pseudogenes, or RetroGenes V9 (Baertsch et al. 2008; Harrow et al. 2012; Pruitt et al. 2014). They do not overlap known protein-coding regions, but some may be unknown protein-coding exons rather than fossils. Frameshifts or premature stop codons are present in 71.3% of the new segments and 72.4% of the non-new ones, suggesting a similar (presumably low) fraction of unknown coding exons.

Ancient fossils were sought in the same way as for TEs, but there is an extra difficulty. While we may find a fossil in the human genome that overlaps an alignment to (say) chimera, it might have encoded a functional protein for most of this evolutionary history, becoming a fossil only recently in the human lineage (Sheetlin et al. 2014). The aligned region of chimera was also required to be noncoding, but it may have independently become a fossil, or simply be an unannotated protein-coding exon. The nonhuman genomes presumably have less thorough gene annotation.

Thus, ancient fossils were checked by manually examining UCSC phyloP graphs showing basewise evolutionary conservation in 100 vertebrates (Pollard et al. 2010). In some cases, there was a pattern of every third base being less conserved, indicating that natural selection conserved the encoded amino acids, for at least part of the history (supplementary fig. 8*A*).

**Fig. 7.**
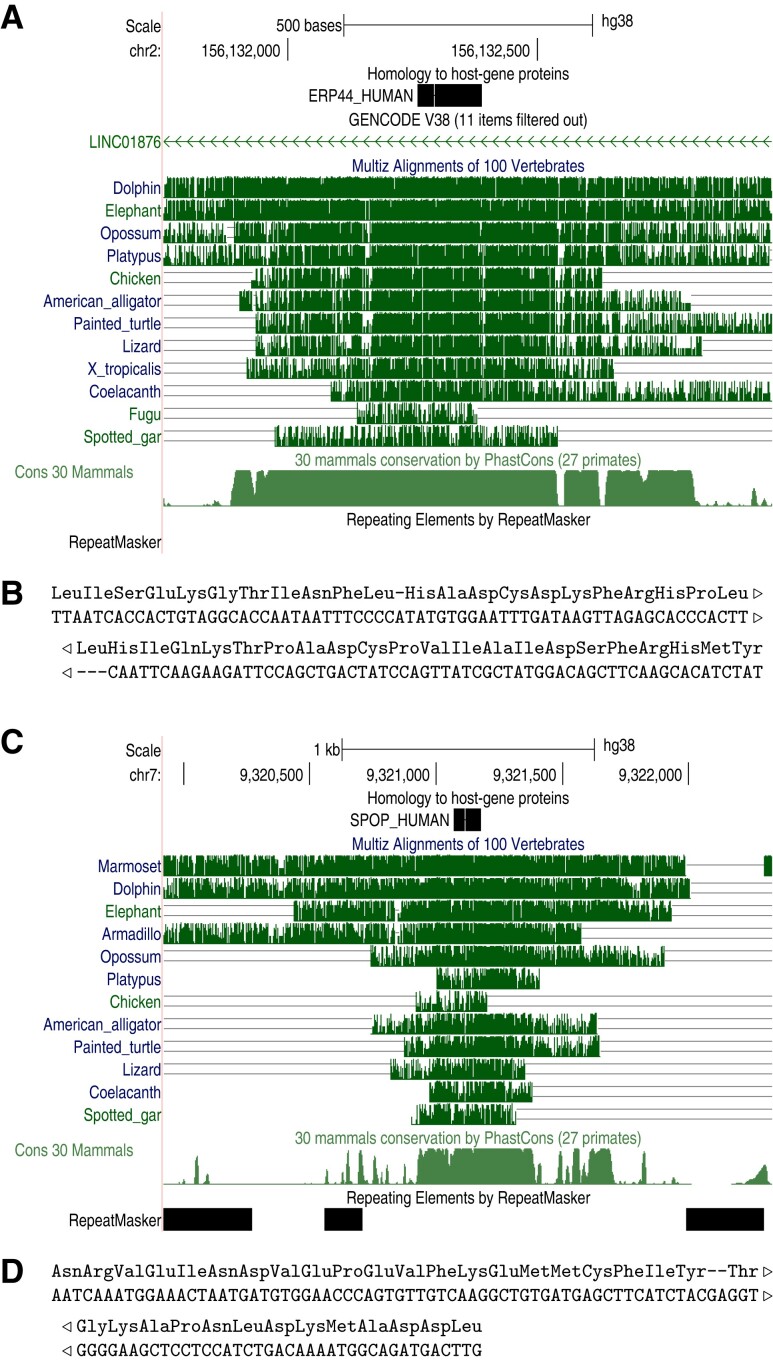
Ancient conserved pseudogenes in the human genome. (*A*) Match between endoplasmic reticulum resident protein 44 and chromosome 2, showing conservation in vertebrates. (*B*) Base-level alignment of the above. (*C*) Match between speckle-type POZ protein and chromosome 7. (*D*) Base-level alignment thereof.

In the end, two strong candidates were found for host-gene-derived fossils predating the last common ancestor of jawed vertebrates (fig. 7). These human regions are aligned to alligator, turtle, coelacanth, and chimera, and are not annotated as protein-coding in any of these genomes. The DNA–protein alignments have frameshifts (fig. 7*B* and *D*), and the basewise conservation does not suggest 3-periodicity (supplementary fig. 8). Their ancient conservation, and strong phastCons conservation in mammals, testifies to their exaptation for some critical but unknown function.

### Conclusions and Prospects

This study greatly increases the number and variety of Paleozoic protein fossils. Fossils of most major TE categories (except Helitrons) are found that predate the amphibian/amniote divergence. The oldest fossils, from both TEs and host genes, predate the last common ancestor of jawed vertebrates. The detection of some TE types in ancestral genomes makes their distribution in vertebrates less patchy, suggesting that ancient vertebrates had a high diversity of TEs that were vertically inherited in some lineages but lost activity in others. There are hints that marine or aquatic vertebrates are prone to horizontal TE transfer (Ivancevic et al. 2018; Zhang et al. 2020; Barreat and Katzourakis, 2021), which might explain the high ancestral diversity. These ancient fossils have strong sequence conservation, indicating exaptation, and some have evidence of regulatory function. Not only TEs but also host-gene fossils were anciently exapted with strong sequence conservation. Ancient fossils can be markers of long-term genome tectonics.

It is hoped that these fossil-finding methods can easily be adapted for future studies. They are especially beneficial for finding TEs in less-studied genomes, reducing reliance on de novo repeat-finding and confusion between low copy-number TEs, multi-gene families, and TE-derived genes (Arkhipova, 2017; Makałowski et al. 2019). The fitting of substitution and gap rates could perhaps be improved: here it was done naively by comparing a genome to known TE proteins. The choice of sequence data for parameter-fitting seems important for finding ancient or unknown types of fossil. Fossil-finding could also be aided by ancestralizing the genome sequence, for example reverting recent substitutions and TE insertions.

One promising application is paleovirology: Few Mesozoic and no Paleozoic viral fossils have been found so far (Barreat and Katzourakis, 2022). If Gypsy-like elements (Metaviridae) or Polintons are counted as viruses, Paleozoic fossils predating ∼350 million years are found here (table 4).

A great challenge is to infer ancient genetic sequences from their fossil fragments, much as ancient organisms are inferred from mineral fossils. This inference might be assisted by LAST’s ability to estimate the probability that each column of a sequence alignment is correct.

## Materials and Methods

The pipeline scripts are available at: https://gitlab.com/mcfrith/protein-fossils.

### Genome Data

Genome sequences and their RepeatMasker annotations were downloaded from UCSC, NCBI, or repeatmasker.org (table 1). The human RepeatMasker annotations are from UCSC’s rmskOutCurrent file (last modified October 28, 2018).

TE protein sequences were taken from the file RepeatPeps.lib in RepeatMasker version 4.1.2-p1. For each nonhuman genome, proteins encoded by host genes were taken from NCBI’s .faa file for that genome. For human, with the aim of getting reliable proteins, non-TE proteins with existence level 1–3 were taken from uniprot_sprot_human.dat in UniProt release 2021_02 (The UniProt Consortium, 2020).

Protein-coding regions of the human genome were taken from the union of wgEncodeGencodeCompV37 and ncbiRefSeq from UCSC (Harrow et al. 2012; Pruitt et al. 2014). For each nonhuman genome, protein-coding regions were obtained from NCBI’s .gff file for that genome.

### Finding Protein Fossils

The DNA/protein substitution and gap rates were found separately for each genome, by comparing it to the TE proteins, using LAST version 1250:

lastdb -q -c myDB RepeatPeps.lib

last-train -P8 --codon -X1 --pid=50

 myDB genome.fa > te.train

The -q option appends a stop symbol * to each protein, which can be matched to (fossil) stop codons (e.g. supplementary fig. S9). The --pid=50 option makes it only use homologies with ≤ 50% amino-acid identity, with the aim of focusing on old fossils. Next, the genome was matched to TE and host-gene proteins:

fasta-nr hostProteins RepeatPeps.lib |

 lastdb -q -c pDB

 

lastal -D1e9 -K0 -m500 -p te.train

  pDB genome.fa > aln.maf

Option -D1e9 sets the significance threshold to one false hit per 10^9^ bp, -K0 omits hits that overlap stronger hits in the genome, and -m500 makes it more slow and sensitive. (With lower values of m, occasionally a host-gene-derived fossil was missed and instead wrongly aligned to a TE protein.) Note that the E-values output by lastal are per-chromosome, whereas the E-values in this article are per-genome.

It turns out the RepeatMasker proteins include exapted genes: they were excluded, by omitting hits to proteins whose names contain _HSgene, _Hsa_, UN-GIN, or _Xtr_eg_tp.

Finally, alignments >10% covered by protein-coding annotation were removed, as were host-protein alignments >10% covered by RepeatMasker TE annotations other than Low_complexity and Simple_repeat.

### Genome Alignments

As described above, new pair-wise genome alignments were made, with the aim of finding orthologous segments and avoiding nonhomologous insertions (fig. 2) as accurately as possible. They were made like this:

lastdb -P8 -uMAM8 gDB genome1.fa

 

last-train -P8 --revsym -D1e9

  --sample-number=5000 gDB genome2.fa > g.train

 

lastal -P8 -D1e9 -m100 -p g.train gDB genome2.fa |

 last-split -fMAF+ > many-to-one.maf

 

last-split -r many-to-one.maf |

 last-postmask > one-to-one.maf

The -uMAM8 and -m100 options make it extremely slow and sensitive (Frith and Noé, 2014). These one-to-one alignments are available at https://github.com/mcfrith/last-genome-alignments.

Next, isolated alignments were removed by defining two alignments to be “linked” if, in both genomes, they are separated by at most 10^6^ bp and by at most five other alignments. Alignments were retained if linked, directly or indirectly, to at least two others.

### Ancient Protein Fossils

A protein fossil was inferred to be ancient if it overlaps an inter-genome alignment. However, spurious overlaps are caused by the DNA–protein or inter-genome alignments overshooting beyond the end of homology: this often happens when the fossil is near a protein-coding exon. Therefore, the set of alignments between two genomes was reduced to those that do not overlap protein-coding annotations in either genome, and then each fossil was considered conserved if at least 30% of it is covered by alignments between those two genomes. This 30% threshold was determined empirically (supplementary fig. S9). There is likely a better way using LAST’s ability to estimate the probability of each column in an alignment.

### Novelty

In table 1, a TE fossil was deemed novel if at most 10% of it is covered by RepeatMasker annotations of TEs, with known “class/family,” that are on the same DNA strand.

In tables 4–6, slightly different criteria were used. A TE fossil was deemed “not new” if it has nonzero overlap with a RepeatMasker genome annotation on the same DNA strand, of the same “class” (DNA, LINE, LTR, etc.).

A host-gene protein fossil was deemed novel if at most 10% of it overlaps same-strand known pseudogenes.

### Nearest genes

The nearest genes were found from among those with NM_ accession numbers in ncbiRefSeqCurated from UCSC.

## Supplementary Material

msac068_Supplementary_Data

## Data Availability

The data underlying this article are available in Zenodo, at https://dx.doi.org/10.5281/zenodo.6417614.
