# Supplementary material for: Paleozoic Protein Fossils Illuminate the Evolution of Vertebrate Genomes and Transposable Elements
Source: Mol Biol Evol. 2022 Mar 28;39(4):msac068. doi: 10.1093/molbev/msac068 (PMC9004415; doi:10.1093/molbev/msac068)
Supplement: msac068_Supplementary_Data [file msac068_supplementary_data.pdf]

Supplement to:
Paleozoic protein fossils illuminate the evolution of vertebrate genomes and transposable elements

Martin C. Frith

2022-02-23

A R2-Hero protein (`HEROTn`, length 1181) vs. human chromosome 15, E-value 3.4e-6 (in table 3 and fig. 5B)

```
486      GlyIleProGlyValProGlyCysLeuGluHisCysGlyValValThrGlnLeuIleArgGluAlaArgGluGlyArgGlySerLeuAlaVal--LeuTrpLeuAspLeuAlaAsnAlaTyrGly
         :::|||||||||   ::::::||||||||||||:::   ::::::   |||   ||||||||||||:::   :::   :::||||||           |||||||||::::::|||
67559763 AGAATTCCAGGCTTCTCTAGTTGCCTGGAGCATAGTAATATGATTTTCCAGCACATTAGGGAAGCAAAAGTAGAACCAGAAAGCCTA------TGTACTGGCTAGATTTTGTTAAT---------
         ArgIleProGlyPheSerSerCysLeuGluHisSerAsnMetIlePheGlnHisIleArgGluAlaLysValGluProGluSerLeu        TyrTrpLeuAspPheValAsn

527      SerIle-ProHisLysLeuValGluMetAlaLeuAlaArgHisHisValProGlyPro--IleLysThrLeuIleMetAspTyrTyrAspSerPheHisLeuArgValThrSerGlySerValThr
                :::   |||:::   |||      ||||||||:::::::::            :::      ::::::      ||||||:::...         :::   :::
67559653 ------GTCACTTAAAATGGAGGAAAAAATCCTAGCAAGATACCAAATA---------GAGTGCAGCAGATGGTAAACCAATATTATGAAGCAATCTTCACAAAATCTGCAGTGTGTCACAAATAT
                SerLeuLysMetGluGluLysIleLeuAlaArgTyrGlnIle           ValGlnGlnMetValAsnGlnTyrTyrGluAlaIlePheThrLysSerAlaValCysHisLysTyr

568      SerGluTrpHisArgLeu-GluLysGlyIleIleThrGlyCysThrIleSerValIleIlePhe 588
         ...:::|||::::::||| ||||||:::||||||||||||::::::   ::::::|||...|||
67559542 ACAAAATGGCAGAGTTTGGGAAAAGAGAATCATAACAGGCCATATGTTGTTAGCAATTTTATTT 67559479
         ThrLysTrpGlnSerLeu GluLysArgIleIleThrGlyHisMetLeuLeuAlaIleLeuPhe
```

B RTE-X protein (`RTEX-16_SK_pol`, length 928) vs. human chromosome 19, E-value 0.00078 (in table 4 and fig. 5E)

```
739      CysTyrLeuMetLeuLysArgLeuSerAspGln--ArgValIleThrSerValAsnTrpAlaPhe-AsnValLysIleLeuLeu-PheArgPheGlyPheGlyHisValTrp-LeuAsnGlnGly
         |||||||||:::|||   ...:::   :::     |||   |||:::   ||||||          |||||||||      ||| ::::::::::::|||   :::|||::: ...||||||:::
30154304 TGTTACCTGATATTAAGCTGTTTTGATGAG---TGAGGCAAATTGCTGTGGTTAAC---------AAATGTAAAACCATCACTTTTATTGATACAGGTTTGTGTATGTGTGAAATTAATCAAAGC
         CysTyrLeuIleLeuSerCysPheAspGlu     ArgGlnIleAlaValValAsn          AsnValLysProSerLeu Tyr***TyrArgPheValTyrVal*** IleAsnGlnSer

779      ValGlySerLysSerValPheLeuSer--GlnPheThrGlnArgIleLysAspCysAlaGlnGlnValTrpHisAspAspThrAlaSerSerProLysLeuArgSerTyrLeuAspPheLysSer
         ||||||            ||||||     ...|||   ||||||...:::   :::      :::   :::|||:::   :::      |||      |||      :::   ::::::...:::
30154417 GTGGGCGATGATGAACAGTTTCTG---TAGAATTCTGTCAAAGGTTAAGAGCTAGTTATTTTTAAGAATGACATGAATCTAGCAGAGACAGTGATTGCCTAAATGACCATTGTAATTACCAATTA
         ValGlyAspAspGluGlnPheLeu     GluPheCysGlnArgLeuArgAlaSerTyrPhe***Glu***HisGluSerSerArgAspSerAspCysLeuAsnAspHisCysAsnTyrGlnLeu

820      SerLeuGluGlnGluLys--TyrLeuSerCysIleHisValPheLysPheArgAlaAlaLeuSerArgLeuArgCysSerSerHisAsnLeuArgIleGluLysGlyArgHisGluAsnValPro
            ||||||   |||     |||...   :::::::::   :::|||:::|||   ||||||:::...||||||      ...|||||||||      ||||||||||||   |||:::
30154539 CTTCTTGAAGCAGAG---AATATATTCACGGCGTGCAGTCCTCTAAATACAGAAAGGCATTAGCCCATTTACGTACTAATGCACATAATTTAGCTCCTGAAAAGGGCAGATGGGAGCACAATGAA
         LeuLeuGluAlaGlu     TyrIleHisGlyValGlnSerSerLysTyrArgLysAlaLeuAlaHisLeuArgThrAsnAlaHisAsnLeuAlaProGluLysGlyArgTrpGluHisAsnGlu

861      LeuAspAsnArgLeuCys-GluTyrCysValGlu--ArgAsnGlnLeuTyrValGluAspGluTyrHisPheIleAlaIleCys 887
            ...||||||   ::: :::|||:::        :::|||      :::::::::|||||||||:::|||::::::   |||
30154661 AGATATAATAGATACCGCCTAATATCATTCC---CTAAGAATATTGGATTCATCAAAGATGAATATTACTTTATGTCAGATTGT 30154741
         ArgTyrAsnArgTyrArg ***TyrHisSer     LysAsnIleGlyPheIleLysAspGluTyrTyrPheMetSerAspCys
```

C Nimb proteins (`Nimb-6_DR_pol`, length 1251, E-value 5.6e-14; `Nimb-12_LMi_pol`, length 1256, E-value 2.5) vs. human chromosome X

```
517      SerTyrArgProIleAlaLeuThrSerHisMetCysLysLeuMetGluLysMetIleThrAspArgLeuGlyTyrHisLeuGluLysAsnAsnPhePheSerProTyrGlnSerGlyPheArgSer
         ||||||:::   |||||||||||||||...   ||||||:::||||||   ||||||   ...   |||   :::   |||||||||||||||   :::|||   |||:::|||||||||:::...
87287425 TCTTATCAAATGATAGCCTTAACTTCATAGCCATGTAAGATGATGGAAGAAATGATTTATTATATTCTGTCATTCTTTCTAGAGAAGAATAATCACCTTTCTCAGTATTAAAGTGGCTTTCAGGCA
         SerTyrGlnMetIleAlaLeuThrSer***ProCysLysMetMetGluGluMetIleTyrTyrIleLeuSerPhePheLeuGluLysAsnAsnHisLeuSerGlnTyr***SerGlyPheGlnAla

559      GlyArgGlyThrMetAspSerValIleAsnLeuGluThrAspIleArgLysAlaLeuThrAsnLysGluThrValValAlaValPhePheAspIleGluLysAlaTyrAspMetLeuTrpLysGlu
            :::   |||:::|||   ||||||   |||   :::|||:::   ||||||:::   |||||||||   ...   :::||||||   |||   ::::::   |||      |||   |||:::
87287551 AATCAATCAACCATAGATGGTGTCATAAGACTGTCTTCTGATGTTAATAAAGCATTCCGTAATAAGGAGTTTTTAAGTGTTGTATTTATAGACAGTGATAGAAATTATAAGAGACTGCTGAAAAAA
         AsnGlnSerThrIleAspGlyValIleArgLeuSerSerAspValAsnLysAlaPheArgAsnLysGluPheLeuSerValValPheIleAspSerAspArgAsnTyrLysArgLeuLeuLysLys

601      GlyLeu 602           1075     ArgGlyLysGlnValThrPheCysTrpValProGlyHisValGlyIleArgGlyAsnGluMetAlaAspArgAlaAlaLysGluAla 1103
         ||||||                        :::|||:::   |||:::|||   |||   |||   :::|||||||||:::||||||||||||   |||:::|||:::|||:::|||
87287677 GGACTA 87287682       87289313 CAAGGCAACAATGTCATTTTTGTGTGGTTCCCAGTTTGTGTAGGTATTAAAGGAAATGAAATGGGAGATAAAGCAGTCAAAAAGGCT 87289399
         GlyLeu                        GlnGlyAsnAsnValIlePheValTrpPheProValCysValGlyIleLysGlyAsnGluMetGlyAspLysAlaValLysLysAla
```

Figure S1: Alignments between TE (transposable element) proteins and DNA. The DNA's translation is shown below it, with `***` for stop codons. `|||` indicates a match, `:::` a positive substitution score, and `...` a zero substitution score. Red color indicates conserved residues in LINE RT domains (Malik *et al.* 1999). This figure was made with `maf-convert` from the LAST package.

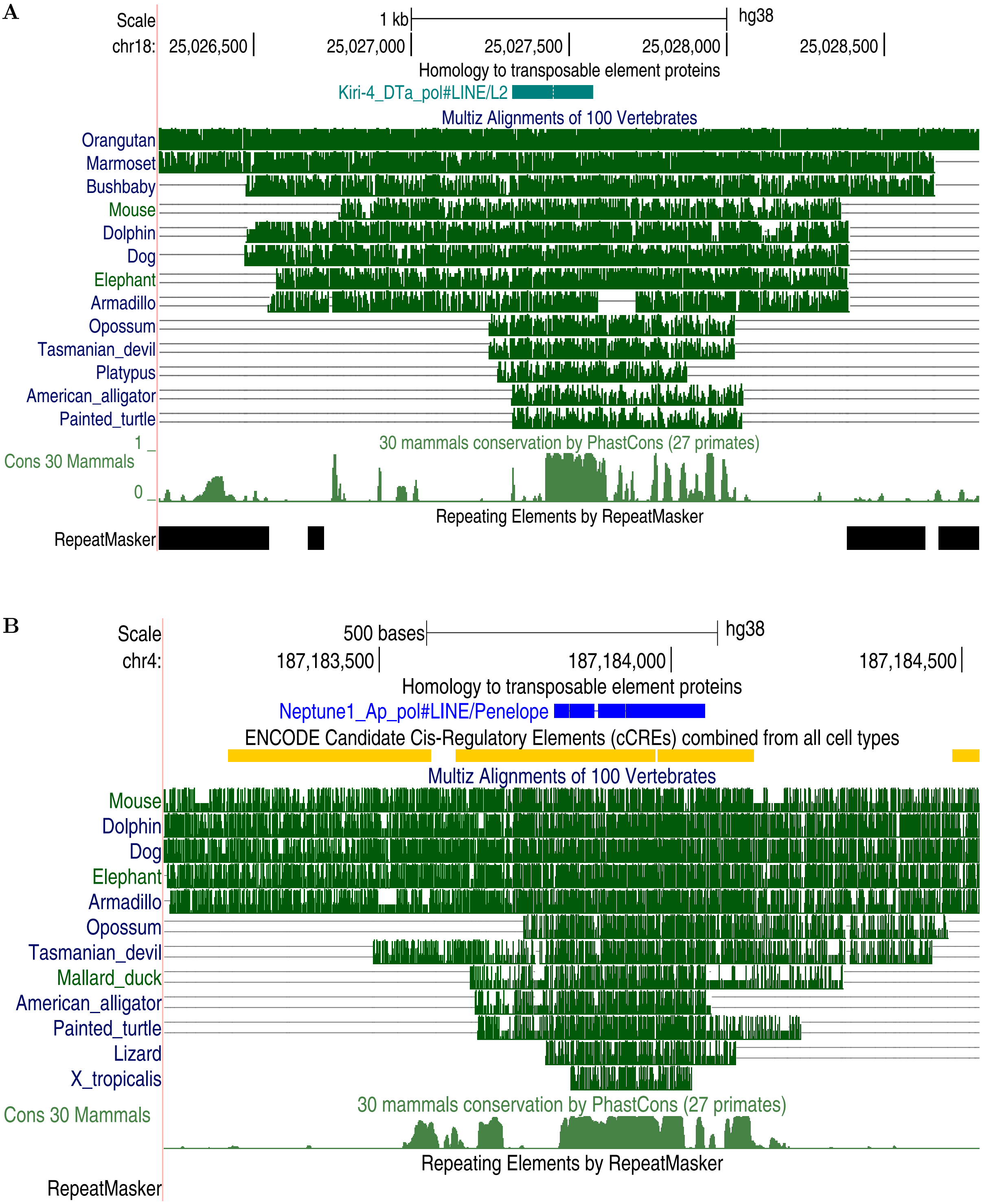

Figure S2: Ancient conserved TE insertions in the human genome. (A) An L2-Kiri protein fossil in chromosome 18 (from table 3). (B) A Penelope-like protein fossil in chromosome 4 (from table 4). Screen shots from `http://genome.ucsc.edu`.

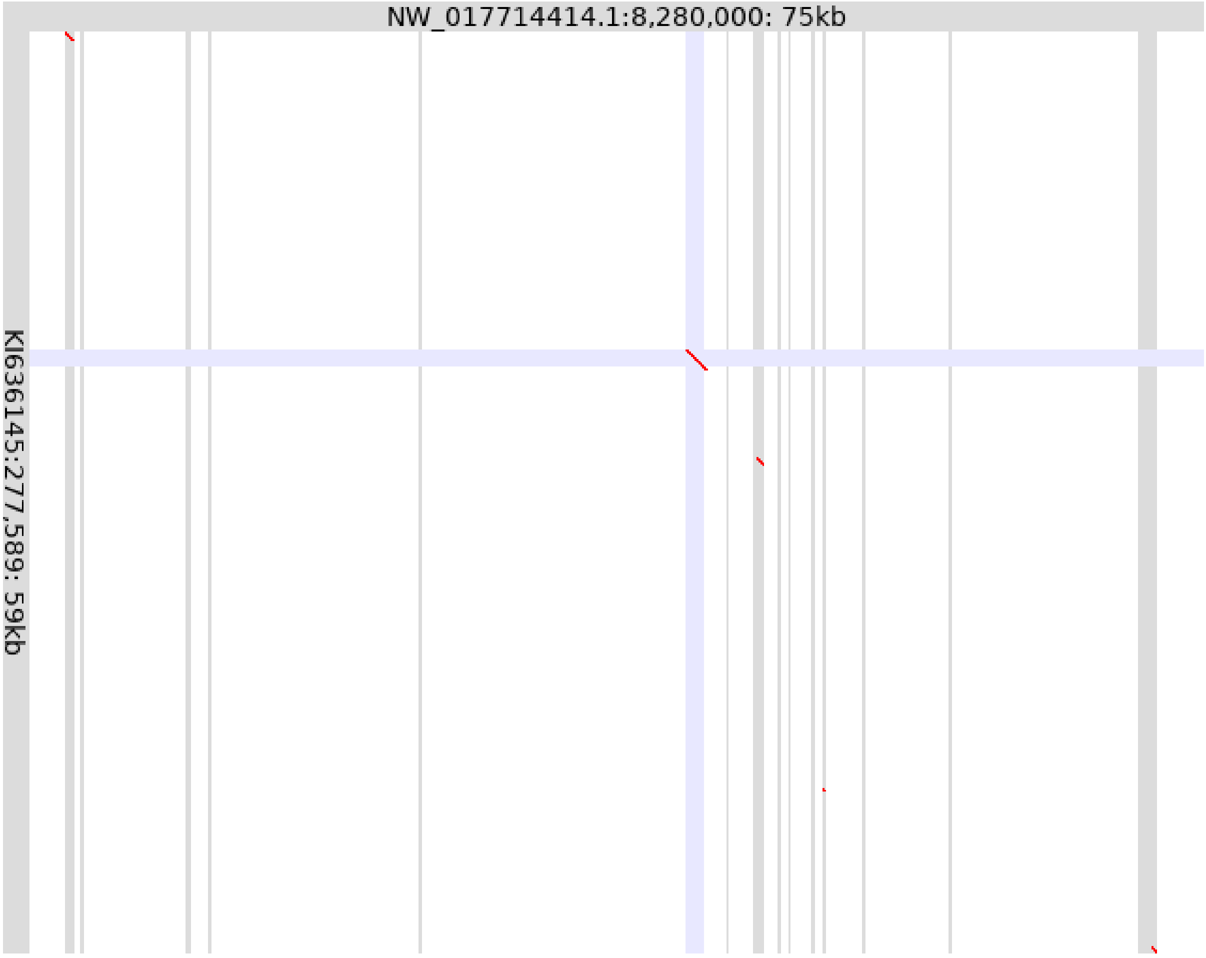

Figure S3: A Crypton insertion that predates the last common ancestor of alligator and chimaera. The red dots show alignments between part of an alligator genome (horizontal) and a chimaera genome (vertical). The vertical blue stripe shows the location of a Crypton protein fossil in the alligator genome. The horizontal blue stripe indicates a Crypton protein fossil in chimaera. The vertical gray lines show protein-coding exons in alligator of the *ATF7IP* gene, which codes for activating transcription factor 7 interacting protein.

```
50      GluAspAspLysAspGluValAsnThrLysLysIleThrLysTrpAlaValAsnIlePheArgGluPheLeuAlaGlnLysAsnMetAspIleAsnPheGluAsnTyrThrAlaThrThrLeuAsn
        ||||||   |||         ||||||   |||   |||   ||||||:::|||:::   ::::::   ||||||.........   |||   :::||||||         :::|||   ||||||
8322962 gaagatgCTAAGAGCATCAAAAACACCCACAAGCAGACTGGCTGGGCAGCTAATCTACTAAAGCAGTGGCTGGCCAAAAATGGCAAGGATCCTAGTTTTGAATTGGTGCCAGTAACTGAACTCAAT
        GluAspAlaLysSerIleLysAsnThrHisLysGlnThrGlyTrpAlaAlaAsnLeuLeuLysGlnTrpLeuAlaLysAsnGlyLysAspProSerPheGluLeuValProValThrGluLeuAsn

92      GluSerLeuArgLeuPheTyrAlaSerValGlnSerThrLysGluGlyGlyGluTyrSerValAlaSerLeuArgSerLeuArgAlaGlyIleAsnArgHisLeu------------------Arg
        :::   ||||||   ||||||   ::::::   :::      :::|||      |||||||||||||||   :::|||:::|||||||||...||||||||||||                  |||
8322836 GATATTTAAGAGAGTTTATTACACAATAAGGAAC---CATGATGGAAATACCTACAGTGTGGCAAGTTATAAGTCCATGCGTGCTGGCTTGAACCGGCACCTTAAAAcgccaccATATAATCGT
        AspIleLeuArgGluPheTyrTyrThrIleArgAsn   HisAspGlyAsnThrTyrSerValAlaSerTyrLysSerMetArgAlaGlyLeuAsnArgHisLeuLysThrProProTyrAsnArg

128     AspValAsnIleIleSerAspThrValPheLysSerSerAsnAlaValPheLysAlaIleMetLysArgTyrArgLysSerGlyLysAspThrSerSerHisHisProArgIleProGluSerAsp
           :::   ::::::   |||      |||   |||...|||   ||||||      ::::::|||      |||      |||||||||   :::   |||||||||   |||         |||
8322713 CAGATTTGCCTAATGAAGGACAAGGAGTTTGCTAGTGCAAACATGGTGTTTGTGAGTGTGTTGAAGATGCTGCGCGTGCAGGGAAAGGATGAAACTCACCACCACCCTCCCATAGCTGCTGAGGAC
        GlnIleCysLeuMetLysAspLysGluPheAlaSerAlaAsnMetValPheValSerValLeuLysMetLeuArgValGlnGlyLysAspGluThrHisHisHisProProIleAlaAlaGluAsp

170     LeuGluLysIleArgCysSerSerAlaLeuSerArgHisAlaPro---ArgLeuValArgLysValTrpPheAspIleGlnLeuCysLeuAlaArgArgGlyArgGluGlyCysArgGluLeuThr
        |||   ||||||:::   |||   :::|||      |||   |||      ||||||   |||||||||||||||...||||||......|||:::||||||||||||      |||:::|||:::
8322587 CTGCGTAAGATTAAGCAGTCTGGAGTGCTGGGGTTGCATAGTCCCCTGGCTTTGGTCAACAAGGTGTGGTTTGATTTGCAGTTGCATTTTGCCAAACGAGGGAGGGAAATCTTACGAGATTTGGCT
        LeuArgLysIleLysGlnSerGlyValLeuGlyLeuHisSerProLeuAlaLeuValAsnLysValTrpPheAspLeuGlnLeuHisPheAlaLysArgGlyArgGluIleLeuArgAspLeuAla

211     MetAlaSerPheSerIleHisArgAspGluGluGlyAlaGluTyrLeuSerLeuSerHisAsnProAspThrLysAsnHisLysThrProAsnAspProHisLysGlnAsnLeuArgGlyPheMet
              :::|||   :::   :::|||:::   |||      |||      ...   :::               ...   |||         ||||||||||||         :::|||   |||
8322461 CCAGATGCCTTTGTTGTTGAGAAGGACAAGAATGGGCGTCGGTATGCTATGTTTAGATGT---------CCTGGCAAAGGGAAAAATGCAGAAGATCCCCATAAa---------aaggggaaaatg
        ProAspAlaPheValValGluLysAspLysAsnGlyArgArgTyrAlaMetPheArgCys         ProGlyLysGlyLysAsnAlaGluAspProHisLys         LysGlyLysMet

253     PheAlaArgProGlyAspProLeuCysProIleGlnSerPheLysLysTyrIleSerLysCysProProAspAlaLysSerPheTyrLeuHisProLys--ArgSerValThrAlaAlaSerGlu-
        :::      ||||||||||||   ||||||:::   |||...      |||...||||||   ||||||:::      :::|||||||||||||||     ::::::   :::   |||    ...
8322353 tATGATATGCCAGGGGACCCAAACTGTCCTGTTTTTCCTTGGAGCTTTATTTGTCTAAGTTGCCTCCTGAGCCCCCTGCTTTTACCTGCATCCT---TTAAAGCTAACTCTAGAGCAGATGCGA
        TyrAspMetProGlyAspProAsnCysProValPheSerLeuGluLeuTyrLeuSerLysLeuProProGluProProAlaPheTyrLeuHisPro     LysAlaAsnSerArgAlaAspAla

294     ---------ValTrpTyrSerArgGluProMetGlyValAsnTyrLeuGlyAlaMetLeuLysLysIleSerGluGluValGlyLeuSerGlnIleTyrThrAsnHisSerLeuArgSerThrAla
                 |||||||||   ||||||||||||||||||||||||||||||:::|||:::   :::||||||      ::::::|||||||||   |||||||||||||||||||||:::|||:::
8322230 GAGCAGCCTGTCTGGTACAAACGGGAGCCTATGGGAGTAAACTACTTAGGTACTATGATGCCCAGAATAAGCGTGGCAGCCAGGCTGTCCCAACGGTATACCAATCATTCTCTCAGAACTACCACC
                 ValTrpTyrLysArgGluProMetGlyValAsnTyrLeuGlyThrMetMetProArgIleSerValAlaAlaArgLeuSerGlnArgTyrThrAsnHisSerLeuArgThrThrThr

333     ValGlyArgLeuSerAspAlaGlyLeuGluSerArgGlnIleMetSerValThrGlyHisArgCysGluSerSerLeuGlnAlaTyrTrpAlaProSerLeuGlnGluArgArgGluTrpSerAsn
        :::      |||...:::|||||||||...   |||:::||||||...|||||||||||||||:::||||||:::...      ||||||      ...            |||   ||||||:::
8322104 ATCCAGCTACTATGTGAAGCAGGACTGGGGCCTAGAGAGATCATGGCAGTGACAGGCCATCGCTCTGAGTCTGCTATTAGACATTACTGGGGAGCTGCAGAAATTCGCTACAGAGCTTGGTCCGAT
        IleGlnLeuLeuCysGluAlaGlyLeuGlyProArgGluIleMetAlaValThrGlyHisArgSerGluSerAlaIleArgHisTyrTrpGlyAlaAlaGluIleArgTyrArgAlaTrpSerAsp

375     IleLeu 376
        |||:::
8321978 ATAATG 8321973
        IleMet
```

Figure S4: Alignment between a Crypton protein (`CryptonA-1_OL_yr`, length 423) and alligator DNA (`NW_017714414.1`). Catalytically essential residues are shown in red. The DNA's translation is shown below it. The DNA's reading frame is essentially intact: the alignment has (perhaps incorrectly) two nearby frameshifts (near protein coordinate 294), which counteract each other to restore the frame, and the intervening DNA is translatable without shifting frame.

```
36     argarghislysgluValSerProGluGluLeuAsnPheLeuGluAspAspLysAspGluValAsnThrLysLysIleThrLysTrpAlaValAsnIlePheArgGluPheLeuAlaGlnLysAsn
       |||||||||      |||:::   |||:::|||      ||||||::::::::::::|||   ||||||:::|||   |||   |||||||||||||||   ::::::   |||   :::|||:::
298913 AGGCGGCACGTTAGCGTGACCGAGGAGGACCTTATTGTCCTTGAGGAGGAACGCAATGAAAAGAACACTCGCAAGCAGACCGACTGGGCAGTAAACATCCTAAAGCAGTGGCTCATTGAGAAAGGC
       ArgArgHisValSerValThrGluGluAspLeuIleValLeuGluGluGluArgAsnGluLysAsnThrArgLysGlnThrAspTrpAlaValAsnIleLeuLysGlnTrpLeuIleGluLysGly

78     MetAspIleAsnPheGluAsnTyrThrAlaThrThrLeuAsnGluSerLeuArgLeuPheTyrAlaSerValGlnSerThrLysGluGlyGlyGluTyrSerValAlaSerLeuArgSerLeuArg
       ...|||   :::||||||      ::::::         ||||||:::   ||||||   ||||||   :::|||...         :::|||   ...||||||::::::|||   :::|||:::|||
298787 CAAGATGAACACTTTGAGGTTATGTCAGTGGAGGAACTTAACGGAGTCTTGAGAGAGTTCTACGGGACTGTGCGCAAT---CATGACGGGAACACGTACAGCATCTCCAGCTACAAGTCCATCCGG
       GlnAspGluHisPheGluValMetSerValGluGluLeuAsnGlyValLeuArgGluPheTyrGlyThrValArgAsn   HisAspGlyAsnThrTyrSerIleSerSerTyrLysSerIleArg

120    AlaGlyIleAsnArgHisLeu------------------ArgAspValAsnIleIleSerAspThrValPheLysSerSerAsnAlaValPheLysAlaIleMetLysArgTyrArgLysSerGly
       ||||||:::||||||||||||                  |||   :::   ::::::   |||      |||   |||:::|||   ||||||      :::   |||      ||||||   |||
298664 GCCGGGTTAAACCGACACCTTAAGATGCCCCCACACCTCCGGCAGATATGCCTGATGCAGGATAAGGAATTCACCAGCGCCAATAACGTCTTCCTGGGTGTGCTCAAGATCCTCCGCAAACAAGGC
       AlaGlyLeuAsnArgHisLeuLysMetProProHisLeuArgGlnIleCysLeuMetGlnAspLysGluPheThrSerAlaAsnAsnValPheLeuGlyValLeuLysIleLeuArgLysGlnGly

156    LysAspThrSerSerHisHisProArgIleProGluSerAspLeuGluLysIleArgCysSerSerAlaLeuSerArgHisAlaProArg---LeuValArgLysValTrpPheAspIleGlnLeu
       ||||||   :::   |||||||||   |||      :::||||||   |||||||||...|||   :::|||      |||:::|||      ||||||   |||||||||||||||:::||||||
298538 AAGGACGAGACCAACcaccacccccccattaatGCTGCTGACCTGCGGAAGATCAGGACATCGGGGGTGCTGGGTCTCCACACCCCCCTCGCTCTGGTCAACAAAGTCTGGTTCGATTTGCAGTTG
       LysAspGluThrAsnHisHisProProIleAsnAlaAlaAspLeuArgLysIleArgThrSerGlyValLeuGlyLeuHisThrProLeuAlaLeuValAsnLysValTrpPheAspLeuGlnLeu

197    CysLeuAlaArgArgGlyArgGluGlyCysArgGluLeuThrMetAlaSerPheSerIleHisArgAspGluGluGlyAlaGluTyrLeuSerLeuSerHisAsnProAspThrLysAsnHisLys
          :::|||:::||||||||||||      |||:::|||         :::|||   |||   ||||||      |||      |||      |||   :::            :::|||   ...
298412 CACTTTGCCAAGCGTGGGCGGGAAATCCTCCGGGACCTCCCTCCAGACGCCTTTGTTATCAAGCGAGACCCCAACGGCCGTCGCTACGCGATGCTGAAATACACCGGCAAAGGGAGAAACCGGGAA
       HisPheAlaLysArgGlyArgGluIleLeuArgAspLeuProProAspAlaPheValIleLysArgAspProAsnGlyArgArgTyrAlaMetLeuLysTyrThrGlyLysGlyArgAsnArgGlu

239    ThrProAsnAspProHisLysGlnAsnLeuArgGlyPheMetPheAlaArgProGlyAspProLeuCysProIleGlnSerPheLysLysTyrIleSerLysCysProProAspAlaLysSerPhe
                ||||||   |||            |||   |||:::      |||||||||      ||||||:::   |||         |||:::||||||   |||||||||      :::|||
298286 ---------GACCCGCTCAAACTG---------GGCCGCATGTACGACATGCCTGGGACATCAACTGCCCTGTTACCTCCCTGGACGTTTACCTCTCTAAGCTACCGCCAGACCCTCCTGCCTTC
                AspProLeuLysLeu         GlyArgMetTyrAspMetProGlyAspIleAsnCysProValThrSerLeuAspValTyrLeuSerLysLeuProProAspProProAlaPhe

281    TyrLeuHisProLysArgSerValThrAla---------AlaSerGluValTrpTyrSerArgGluProMetGlyValAsnTyrLeuGlyAlaMetLeuLysLysIleSerGluGluValGlyLeu
       ||||||||||||   :::   ...               ...      |||||||||   ||||||||||||||||||||||||||||||   |||:::   :::||||||         ...|||
298178 TACCTTCACCCCCTCAAGCTGACTCCCGAGCAGATCCAGGAACAGTCCGTCTGGTACAAGCGGGAGCCGATGGGAGTGAACTATCTGGGCAGTATGATGCCTCGGATCAGCATTGCTGCCAGGCTC
       TyrLeuHisProLeuLysLeuThrProGluGlnIleGlnGluGlnSerValTrpTyrLysArgGluProMetGlyValAsnTyrLeuGlySerMetMetProArgIleSerIleAlaAlaArgLeu

320    SerGlnIleTyrThrAsnHisSerLeuArgSerThrAlaValGlyArgLeuSerAspAlaGlyLeuGluSerArgGlnIleMetSerValThrGlyHisArgCysGluSerSerLeuGlnAlaTyr
       ||||||   |||||||||||||||||||||:::|||:::|||      |||:::||||||||||||::::::|||:::||||||:::   ||||||||||||:::|||||||||:::      |||
298052 TCCCAGAGATACACCAACCATTCCCTGAGAACTACAACTGTCCGCCTCCTGTGTGACGCGGGTCTGGGAGCCCGGGAGATCATGGCACTCACTGGCCACCGATCCGAATCCAGTATCAGAAACTAC
       SerGlnArgTyrThrAsnHisSerLeuArgThrThrThrValArgLeuLeuCysAspAlaGlyLeuGlyAlaArgGluIleMetAlaLeuThrGlyHisArgSerGluSerSerIleArgAsnTyr

362    TrpAlaProSerLeuGlnGluArgArgGluTrpSerAsnIleLeu 376
       |||      :::      :::   |||   ||||||::::::|||
297926 TGGGGAGCTGCGGAGTTTCAGTACCGAGCCTGGTCCGACATGCTG 297882
       TrpGlyAlaAlaGluPheGlnTyrArgAlaTrpSerAspMetLeu
```

Figure S5: Alignment between a Crypton protein (`CryptonA-1_OL_yr`, length 423) and chimaera DNA (`KI636145`). Catalytically essential residues are shown in red. The DNA's translation is shown below it. The DNA's reading frame is intact.

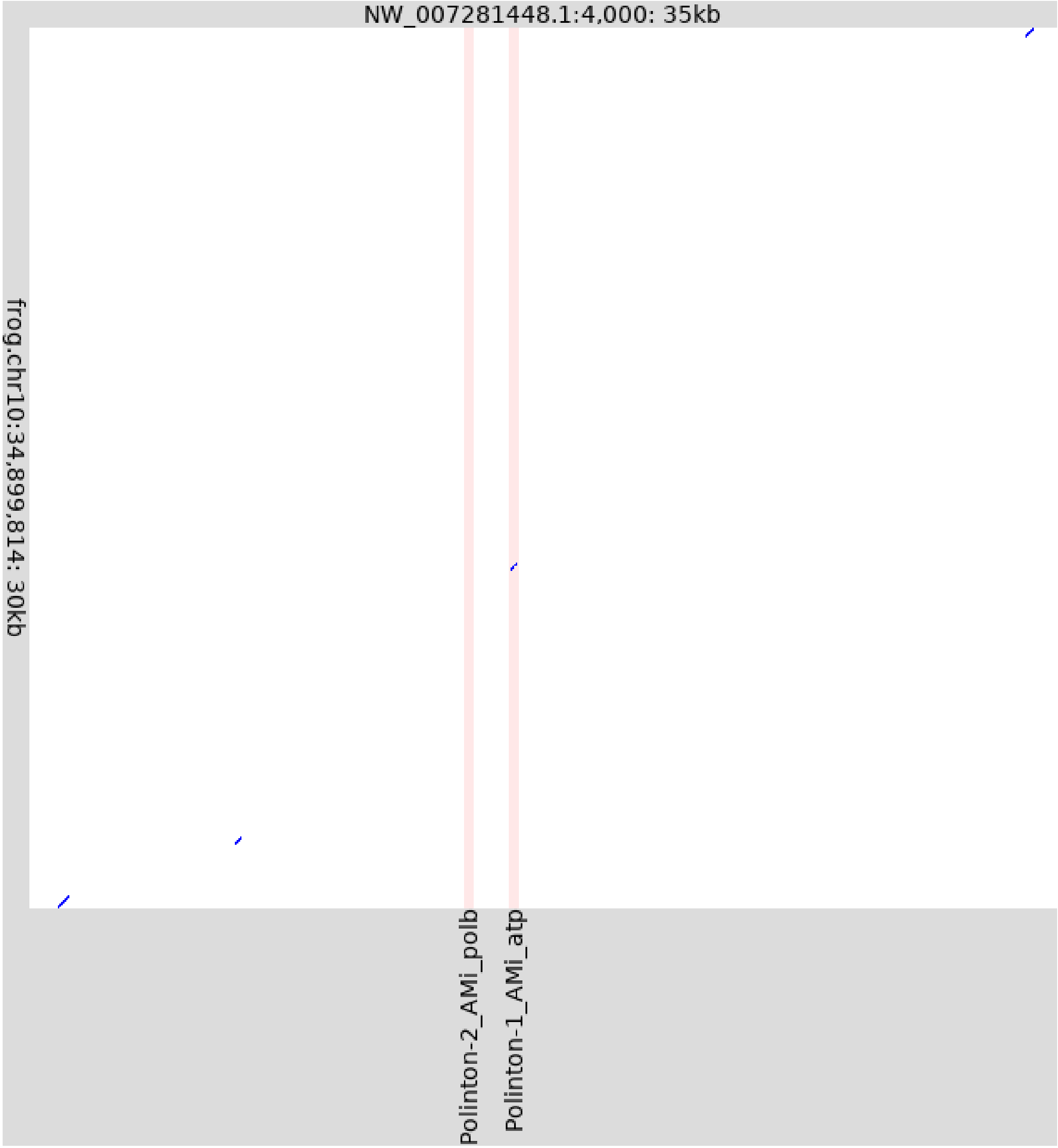

Figure S6: A Polinton insertion that predates the last common ancestor of turtle and frog. The blue dots show alignments between part of a turtle genome (horizontal) and a frog genome (vertical). The red stripes show turtle DNA segments that have homology to Polinton proteins. The atp homology is listed in table 4.

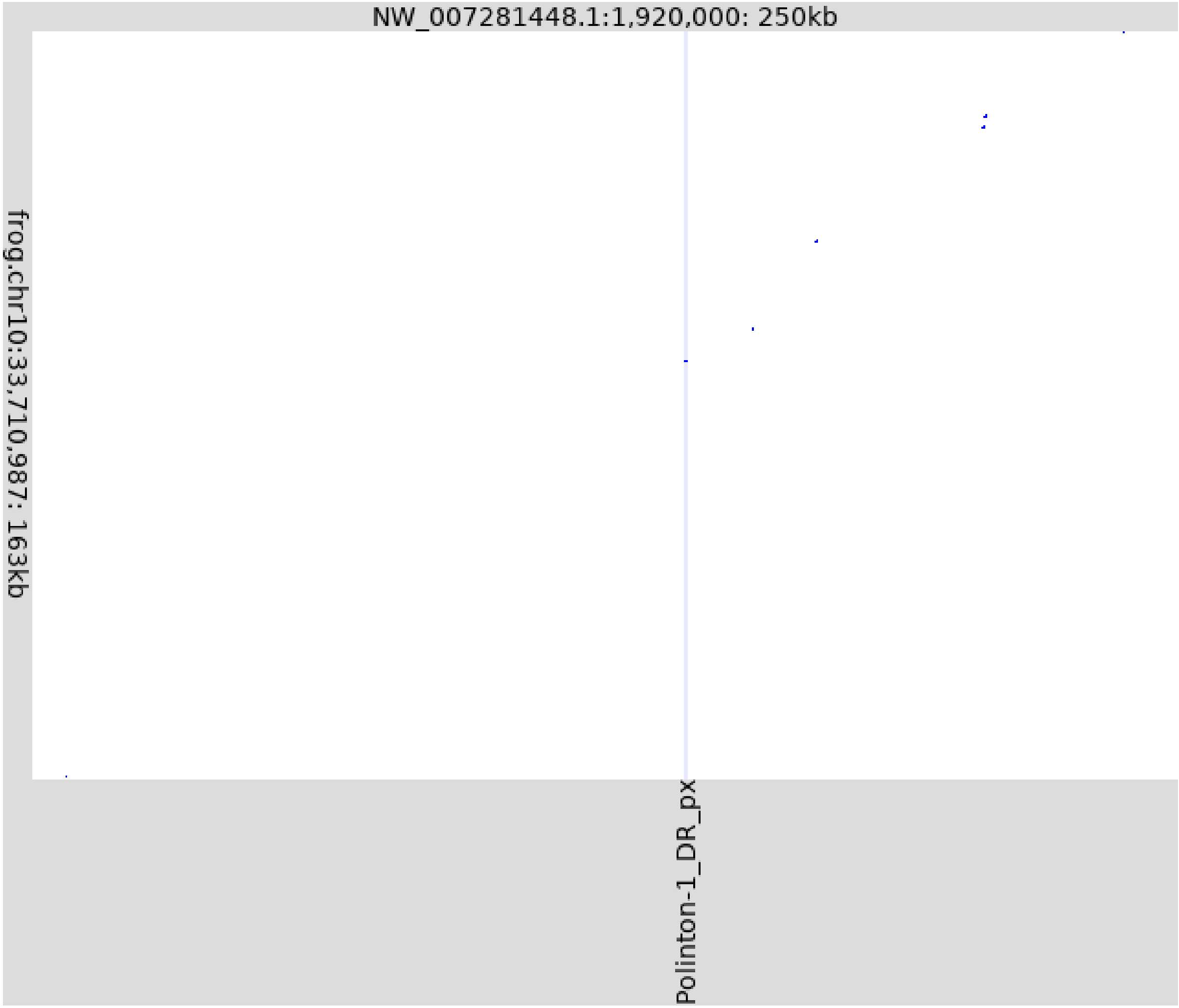

Figure S7: A Polinton insertion that predates the last common ancestor of turtle and frog. The blue dots show alignments between part of a turtle genome (horizontal) and a frog genome (vertical). The blue stripe shows the location of a Polinton protein fossil in turtle (table 4).

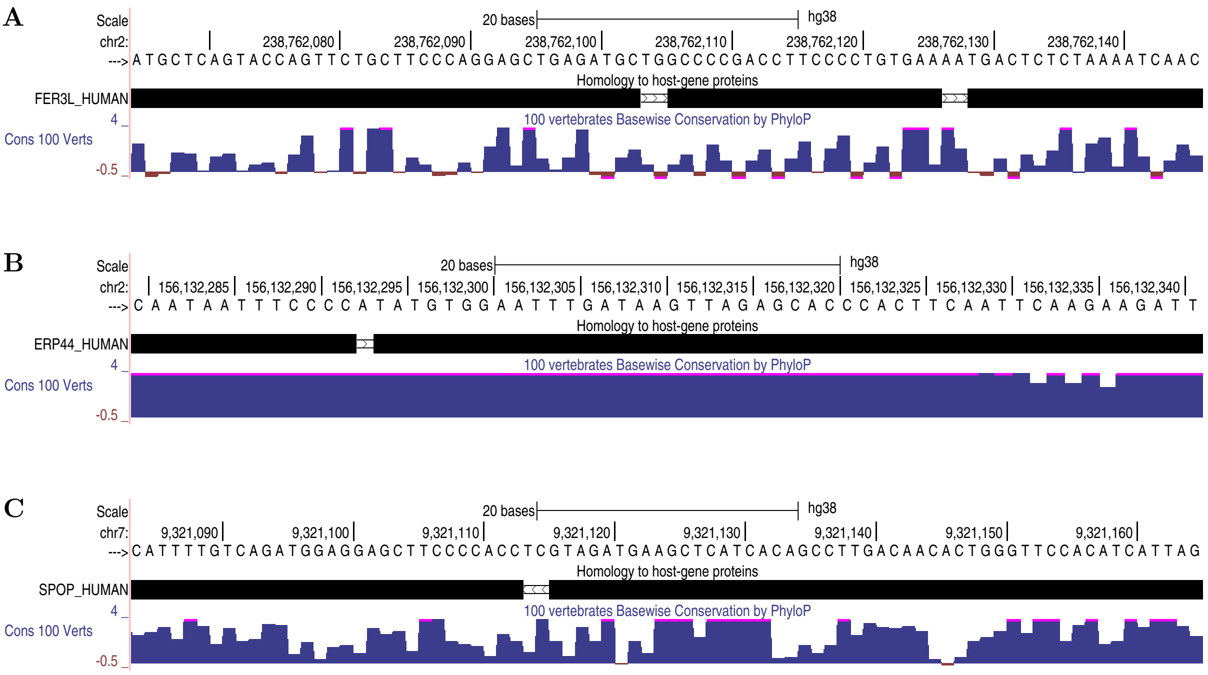

Figure S8: Host-gene-derived protein fossils in the human genome, and their basewise evolutionary conservation in vertebrates. (A) A protein fossil that has two frameshifts (shown as gaps in the homology), but its basewise conservation has a pattern of every 3rd base being less conserved (and this pattern shifts frame at the frameshifts). This implies the DNA evolved under natural selection to preserve an encoded protein for much of its history in vertebrates, and lost protein-coding function at some point in the lineage leading to humans. (B) Basewise conservation of the same protein fossil as in fig. 7 A, B. (C) Basewise conservation of the same protein fossil as in fig. 7 C, D (opposite DNA strand).

```
1241      MetSerPheValGlyThrTrpMetLysLeuGluIleIleIleLeuSerLysLeuSerGlnGluGlnLysThrLysHisArgIlePheSerLeuIleGlyGlyAsn*** 1276
          :::   |||         ::::::|||:::|||:::||||||||||||||||||||||||:::|||||||||||||||::::::||||||||||||||||||||||||
192147655 ATTAATTTTTTTAACGTTTGTTGAAAATGGAAACCATCATTCTCAGCAAACTGTCACAAGGACAAAAAACCAAACACTGCATGTTCTCACTCATAGGTGGGAATTGA 192147548
          IleAsnPhePhe***ArgLeuLeuLysMetGluThrIleIleLeuSerLysLeuSerGlnGlyGlnLysThrLysHisCysMetPheSerLeuIleGlyGlyAsn***
                             =========================================================================================
```

Figure S9: Alignment between an L1 LINE protein (`UN-L1PA2_pol`, length 1276 including stop symbol shown as `***`) and human chromosome 2. The DNA's translation is shown below it. The DNA region underlined by `=====` is annotated as an L1PA5 element by RepeatMasker. The red DNA region is aligned to the alligator genome. L1PA5 is a relatively young, mammal-specific type of L1, and the high identity of the DNA-to-protein alignment also suggests a young age. The overlap with the human-alligator alignment is likely due to alignment overshoot beyond the end of homology. The human-alligator alignment covers 29/108 bp (27%).

References

Malik HS, Burke WD, and Eickbush TH. 1999. The age and evolution of non-LTR retrotransposable elements. *Mol Biol Evol.* 16(6):793–805.
